# Incidence and Predictors of Worsening Renal Function in Edoxaban-Treated Atrial Fibrillation Patients Within ETNA-AF-Europe Registry

**DOI:** 10.1016/j.jacadv.2024.100880

**Published:** 2024-03-11

**Authors:** Marianne Gwechenberger, Gonzalo Barón-Esquivias, Tim A.C. de Vries, Jolanta M. Siller-Matula, Marius C. Manu, José A.G. Souza, Sebastian Wienerroither, Ladislav Pecen, Joris R. de Groot, Raffaele De Caterina, Paulus Kirchhof, ETNA-AF-Europe Investigators

**Affiliations:** a Department of Cardiology, Medical University of Vienna, Vienna, Austria; b Virgen del Rocio University Hospital, University of Seville, Seville, Spain; c Amsterdam University Medical Centers, University of Amsterdam, Amsterdam, the Netherlands; d Amsterdam Cardiovascular Sciences, Heart Failure and Arrhythmias, Amsterdam, the Netherlands; e Department of Cardiology, Rijnstate Hospital, Arnhem, the Netherlands; f Daiichi Sankyo Europe GmbH, Munich, Germany; g Daiichi Sankyo Austria GmbH, Vienna, Austria; h Institute of Computer Science of the Czech Academy of Sciences, Prague, Czech Republic; i Department of Immunochemistry Diagnostics, University Hospital Pilsen, Pilsen, Czech Republic; j Cardiology Division, University of Pisa, Pisa, Italy; k Fondazione VillaSerena per la Ricerca, Pescara, Italy; l Institute of Cardiovascular Sciences, University of Birmingham, Birmingham, UK; m University Heart and Vascular Centre Hamburg, University Medical Centre Hamburg Eppendorf, Hamburg, Germany; n German Center for Cardiovascular Sciences (DZHK) Partner Site Hamburg/Kiel/Lübeck, Hamburg/Kiel/Lübeck, Germany

**Keywords:** atrial fibrillation, direct acting oral anticoagulant, edoxaban, ETNA-AF-Europe, worsening renal function

## Abstract

**Background:**

Managing patients with atrial fibrillation (AF) and worsening renal function (WRF) remains a clinical challenge due to the need of dose adjustment of non-vitamin K antagonist oral anticoagulants.

**Objectives:**

To determine the incidence of WRF in patients with AF treated with edoxaban, the association of WRF with clinical outcomes, and predictors of WRF and clinical outcomes in these patients.

**Methods:**

This is a subanalysis of the Edoxaban Treatment in routiNe clinical prActice for patients with non-valvular Atrial Fibrillation in Europe study (NCT02944019), an observational study of edoxaban-treated patients with AF. WRF was defined as a ≥25% reduction in creatinine clearance between baseline and 2 years.

**Results:**

Of the 9,054 patients included (69% of the total 13,133 enrolled), most did not experience WRF (90.3%) during the first 2 years of follow-up. WRF occurred in 9.7% of patients. Patients with WRF had significantly higher rates of all-cause death (3.88%/y vs 1.88%/y; *P* < 0.0001), cardiovascular death (2.09%/y vs 0.92%/y; *P* < 0.0001), and major bleeding (1.51%/y vs 0.98%/y; *P* = 0.0463) compared with those without WRF. Rates of intracranial hemorrhage (0.18%/y vs 0.18%/y) and of any stroke/systemic embolic events were low (0.90%/y vs 0.69%/y; *P* = 0.3161) in both subgroups. The strongest predictors of WRF were a high CHA_2_DS_2_-VASc score, high baseline creatinine clearance, low body weight, and older age. Most predictors of WRF were also predictors of clinical outcomes.

**Conclusions:**

WRF occurred in approximately 10% of edoxaban-treated AF patients. Rates of death and major bleeding were significantly higher in patients with WRF than without. Stroke events were low in both subgroups.

Atrial fibrillation (AF) is associated with an increased risk of renal dysfunction.^1^ Patients with AF and moderate-to-severe chronic kidney disease (CKD) are at a higher risk of systemic thromboembolism, stroke, and bleeding compared with those without renal disease; decreasing renal function in patients with CKD is associated with a parallel increasing thromboembolic risk.^2^^,^^3^ Indeed, a crude event rate of 23% per year for worsening renal function (WRF) has been reported in patients with AF taking vitamin K antagonists (VKAs), compared with ∼19%, ∼11%, and ∼15% in patients taking apixaban, dabigatran, and rivaroxaban, respectively.^4^

The availability of real-world evidence complements data from randomized controlled trials, but routine care data on WRF in patients using non-vitamin K antagonist oral anticoagulants (NOACs) are still limited. Combined with the paucity of data on dose adjustments during follow-up in clinical practice and the fact that renal function is a dose reduction criterion for several NOACs, an important challenge remains as to how patients whose renal function worsens over time should be treated, and what the prognostic consequences of WRF are. Thus far, no studies have explored the incidence of WRF and the association between such change in renal function and clinical outcomes in patients treated with edoxaban.

The objectives of this subanalysis of the Edoxaban Treatment in routiNe clinical prActice for patients with non-valvular Atrial Fibrillation in Europe (ETNA-AF-Europe) study are a) to assess change in renal function over time by determining the proportion of edoxaban-treated patients with AF and WRF over a 2-year period, b) to investigate the differences in clinical outcomes in patients who developed WRF compared to those without WRF over a 2-year period, c) to identify predictors of WRF and of clinical outcomes in such patients with AF, and d) to calculate the proportion of patients who had dose reduction if creatinine clearance (CrCl) levels fell <50 mL/min (and vice versa to full dose if renal function improved) over a 2-year period.

## Methods

ETNA-AF-Europe (Clinicaltrials.gov: NCT02944019) is a multinational, multicenter, post-authorization, prospective cohort study conducted in 825 sites in 10 European countries (Austria, Belgium, Germany, Ireland, Italy, the Netherlands, Portugal, Spain, Switzerland, and the United Kingdom).^5^^,^^6^ Patients with AF (re-)confirmed by electrical tracing within the last year were asked to participate if they were treated with edoxaban in doses approved for use in stroke prevention. All participants were requested to attend return visits at the outpatient clinic annually for 4 subsequent years. The current study is based on the first 2 years of follow-up. At each visit, a venous blood sample was collected for several analyses, including measurement of serum creatinine. The study was approved by the Institutional Review Boards and Independent Ethics Committees for all participating centers in compliance with the Declaration of Helsinki and Guidelines for Good Pharmaco-epidemiological Practice. All participants provided written informed consent.

One- and 2-year follow-up outcomes of the 13,133 enrolled patients treated with edoxaban have been previously published.^7^^,^^8^ In this exploratory analysis, we used the last available visit with non-missing information to fill-in missing data at the second visit (last observation carried forward). Patients were excluded from the analysis if data to calculate CrCl were not available for at least 1 of the follow-up time points of 1 year and 2 years. As a uniformly accepted definition for WRF is not currently available, WRF was defined as ≥25% reduction in CrCl from baseline during the 2-year follow-up, similar to that previously used in the Randomized Evaluation of Long-Term Anticoagulation Therapy (RE-LY) trial.^9^

Clinical outcomes in patients with and without WRF were reported, and additional analyses performed taking into consideration the baseline and 2-year CrCl levels. In these additional analyses, patients were categorized according to their CrCl levels as follows:

A) Patients who had poor renal function throughout the study (CrCl constantly ≤50 mL/min [with and without WRF] at baseline and at 2-year follow-up)
B) Patients with decline of renal function (CrCl decline from >50 to ≤50 mL/min [with and without WRF] during the 2-year follow-up)
C) Patients who maintained renal function (CrCl constantly >50 mL/min [with and without WRF] throughout the study [CrCl constantly >50 mL/min without WRF: reference category]), and
D) Patients whose renal function improved (CrCl improvement from ≤50 mL/min to >50 without WRF) during the 2-year follow-up.

### Study outcomes

This study investigated:

• Renal function over time (CrCl at baseline and at 2 years)
• Number of patients with and without WRF
• Clinical outcomes of death (all-cause and cardiovascular [CV)] death); stroke (any stroke/systemic embolic event [SEE], and ischemic stroke); and bleeding (major bleeding and intracranial hemorrhage [ICH]) in patients with and without WRF
• Clinical outcomes of death (all-cause and CV death); stroke (any stroke/SEE, and ischemic stroke); and bleeding (major bleeding and ICH) according to CrCl categories in patients with WRF
• Predictors of WRF and predictors of clinical outcomes
• The proportion of patients for whom their edoxaban dose was adjusted in accordance with the labeled dosing recommendations during the 2 years of follow-up.

### Statistical analysis

Baseline characteristics are summarized as frequencies (n and percentages), mean value ± standard deviation. The chi-square test and Wilcoxon 2-sample test or Kruskal-Wallis test for more than 2 groups were used to compare 2 subgroups with vs without WRF (including the 7 subgroups) and determine statistical significance for categorical and continuous data, respectively. Annualized event rates (%/y) with 95% confidence CIs (based on a normal approximation to the Poisson distribution), related hazard ratios (HRs) and their 95% CIs for the 2 subgroups with vs without WRF (including the 7 subgroups) are presented for all clinical outcomes.

For the CrCl calculation, the Cockcroft–Gault equation^10^ was used for consistency with the summary of product characteristics of edoxaban, which lists moderate or severe renal impairment (CrCl 15-50 mL/min) as a dose reduction criterion.^11^ For the data recategorization, the ‘CrCl constantly >50 mL/min without WRF’ subcategory was used as the reference group to generate the clinical outcomes. Statistical significance was determined using the aforementioned statistical tests and multivariable logistic regression using a stepwise selection (67 variables were considered as potential predictors). At each step, the candidate predictor was chosen based on *P* values, and a *P* value threshold of ≤0.05 (as estimated with the Wald-test) was used to limit the total number of variables included in the final model.

Multivariable logistic regression model stepwise selection was used to identify predictors of WRF. Odds ratios with 95% Wald confidence limits were reported. Age-adjusted Cox model was used to identify predictors of outcome events, with HRs and 95% CIs reported.

Dosing adjustments data are presented as percentages (ie, proportion of patients in whom physicians had followed the edoxaban label for standard dosing or dose reductions at baseline, and at follow-up in this substudy).

## Results

### Baseline characteristics

Of the 13,133 AF patients in the ETNA-AF-Europe study who were included in the 2-year follow-up analysis, 9,054 (69%) with CrCl data recorded at baseline and at 1- or 2-year follow-up were included. Of those patients with sufficient data available, the majority did not experience WRF (90.3%) during the 2 years of follow-up.

Patients who experienced WRF (9.7%) were older (75.8 vs 73.7 years, *P* < 0.0001), more often frail, and had more underlying comorbidities, such as diabetes mellitus (DM), hypertension, valvular disease, and heart failure (HF; Table 1). Of interest, baseline CrCl levels were similar between patients with and without WRF across the different renal function categories (Table 1).

**Table 1:** Baseline Characteristics of Edoxaban-Treated Patients With Atrial Fibrillation With and Without Worsening Renal Function During a 2-Year Follow-Up Period

	With WRF [n = 880 (9.7%)]	Without WRF [n = 8,174 (90.3%)]	*P* Value
Edoxaban dose at baseline			
60 mg, OD	624 (70.9)	6,211 (76.0)	0.0009
30 mg, OD	256 (29.1)	1,963 (24.0)	
Overall adherence to SmPC			
Patients correctly dosed with edoxaban dose at baseline	722 (82.0)	6,817 (83.4)	0.3069
Non-recommended edoxaban dose at baseline	158 (18.0)	1,357 (16.6)	
Male	427 (48.5)	4,664 (57.1)	<0.0001
Age [y]	75.8 ± 9.1	73.7 ± 9.3	<0.0001
Weight [kg]	78.5 ± 17.2	80.8 ± 17.1	<0.0001
Body mass index [kg/m^2^]	28.1 ± 5.3	28.1 ± 5.1	0.5162
Serum creatinine (mg/dL)	0.95 ± 0.34	1.02 ± 0.30	<0.0001
Recalc. CrCl^a^ (CG formula) [mL/min] baseline	76.0 ± 33.4	73.9 ± 29.5	0.3627
≥80	339 (38.5)	2,900 (35.5)	0.3106
50-80	355 (40.3)	3,579 (43.8)	
30-50	162 (18.4)	1,501 (18.4)	
15-30	24 (2.7)	193 (2.4)	
<15	0 (0.0)	1 (0.0)	
Recalc. CrCl^a^ (CG formula) [mL/min] 2 y follow-up (LOCF)	48.4 ± 22.4	74.1 ± 30.2	<0.0001
Recalc. CHA_2_DS_2_-VASc^b^	3.7 ± 1.4	3.2 ± 1.4	<0.0001
Recalc. mod. HAS-BLED^c^	2.7 ± 1.1	2.6 ± 1.1	0.0084
Type of atrial fibrillation			
Paroxysmal	411 (46.8)	4,325 (53.0)	0.0038
Persistent	241 (27.4)	2,010 (24.6)	
Long-standing persistent	21 (2.4)	204 (2.5)	
Permanent	206 (23.4)	1,621 (19.9)	
Perceived frailty	161 (18.3)	921 (11.3)	<0.0001
Medical history			
Diabetes mellitus	248 (28.2)	1,822 (22.3)	<0.0001
Hypertension	736 (83.6)	6,308 (77.2)	<0.0001
Heart failure (derived)	198 (22.5)	1,153 (14.1)	<0.0001
Peripheral artery disease	32 (3.6)	279 (3.4)	0.7299
Coronary heart disease	198 (22.5)	1,738 (21.3)	0.3949
Ischemic stroke	58 (6.6)	499 (6.1)	0.5685
Any bleeding	32 (3.6)	274 (3.4)	0.6575
Valvular disease	188 (21.4)	1,323 (16.2)	<0.0001

Data are presented as mean ± standard deviation for continuous variables or as number (%) for categorical variables.

CG = Cockcroft-Gault; CrCl = creatinine clearance; INR = international normalized ratio; LOCF = last observation carried forward; OD = once daily; SmPC = summary of product characteristics; WRF = worsening renal function.

a CrCl was estimated by Cockcroft-Gault formula.

b Not including complex vascular plaque, and the score was based on derived heart failure.

c Not including labile INR, alcohol use was defined as ≥1 unit/d, and defining the presence or absence of renal or hepatic disease was left to the discretion of the physician.

Significantly less patients with than without WRF received edoxaban 60 mg once daily than edoxaban 30 mg once daily (70.9% and 76.0% vs 29.1% and 24.0%, respectively; *P* = 0.0009). Mean weight (78.5 kg vs 80.8 kg; *P* < 0.0001), CHA_2_DS_2_-VASc score (3.7 vs 3.2; *P* < 0.0001), and HAS-BLED (Hypertension, Abnormal renal/liver function, Stroke, Bleeding history or predisposition, Elderly, Drugs/alcohol concomitantly) score (2.7 vs 2.6; *P* = 0.0084) were significantly different in patients with WRF than those without (Table 1). Patients with WRF also had more advanced forms of AF, that is, persistent and permanent AF (Table 1).

Data recategorized by CrCl levels also showed statistically significant differences between the various CrCl categories in many of the baseline characteristics, including age, weight, body mass index, CHA_2_DS_2_-VASc, and HAS-BLED scores (Supplemental Table 1).

### Clinical outcomes in patients with and without WRF

**Figure 1:**
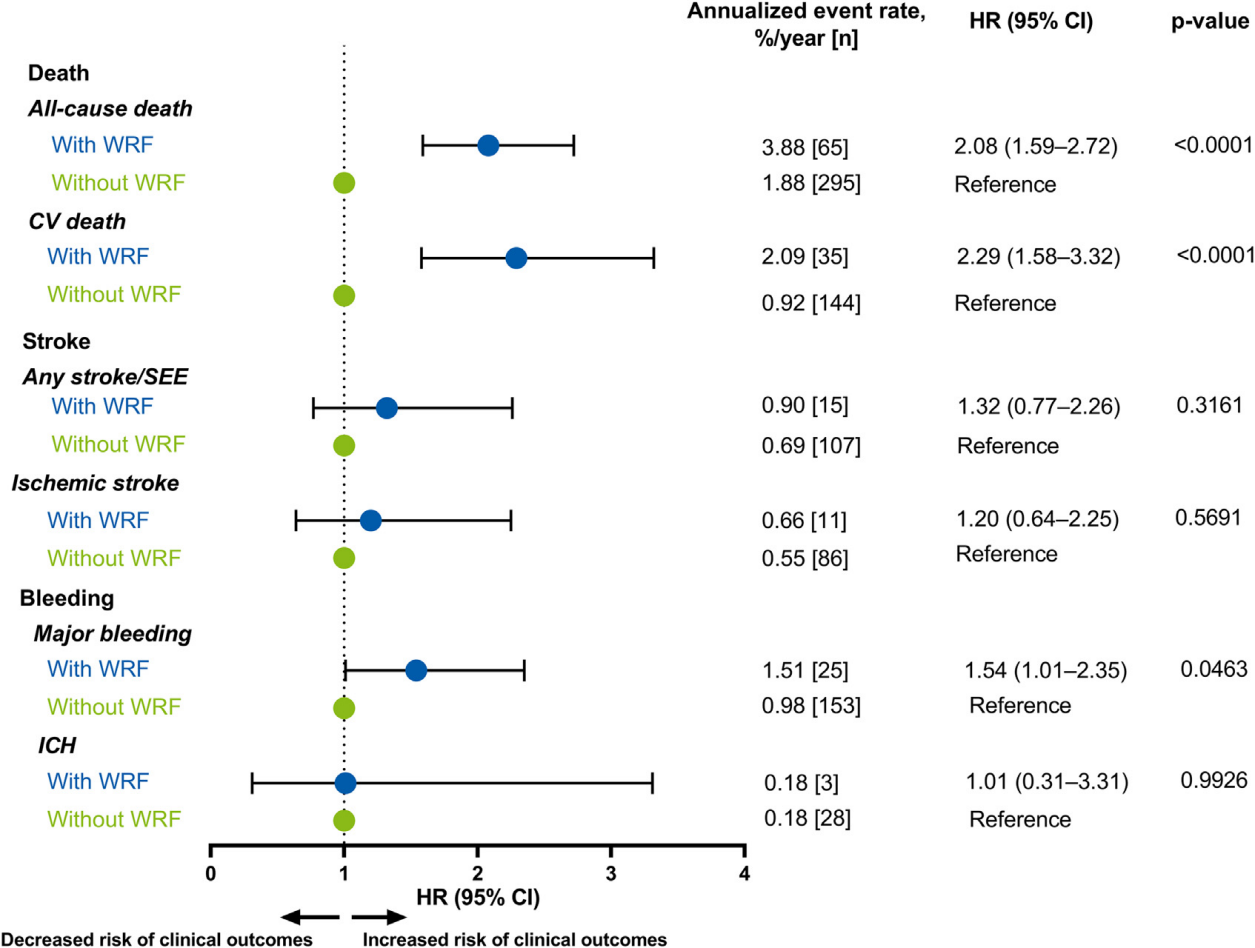
**Unadjusted Association Between Worsening Renal Function and Clinical Outcomes** Forest plot illustrating the association between WRF status and clinical outcomes as modeled by univariable Cox proportional hazard models. Data are represented as annualized event rates, %/y (n); ‘Without WRF’ was used as the reference comparator for each of the clinical outcomes. AF = atrial fibrillation; CI = confidence interval; CV = cardiovascular death; HR = hazard ratio; ICH = intracranial hemorrhage; SEE = systemic embolic event; WRF = worsening renal function.

Patients with WRF had significantly higher annualized event rates of all-cause death (3.88%/y vs 1.88%/y; HR: 2.08 [95% CI: 1.59-2.72]; *P* < 0.0001) and CV death (2.09%/y vs 0.92%/y, 2.29 [95% CI: 1.58-3.32]; *P* < 0.0001) compared with patients without WRF (Figure 1). Annualized rates of major bleeding were significantly higher in patients with WRF than those without (1.51%/y vs 0.98%/y; HR: 1.54 [95% CI: 1.01-2.35]; *P* = 0.046; Figure 1). ICH rates were low (0.18%/y vs 0.18%/y, respectively) in both subgroups (HR: 1.01 [0.31-3.31]; *P* = 0.993; Figure 1). Annualized rates of any stroke/SEE and ischemic stroke were low in patients with WRF, but numerically higher compared with those without WRF (Figure 1).

### Clinical outcomes according to CrCl categories in patients with WRF

**Figure 2:**
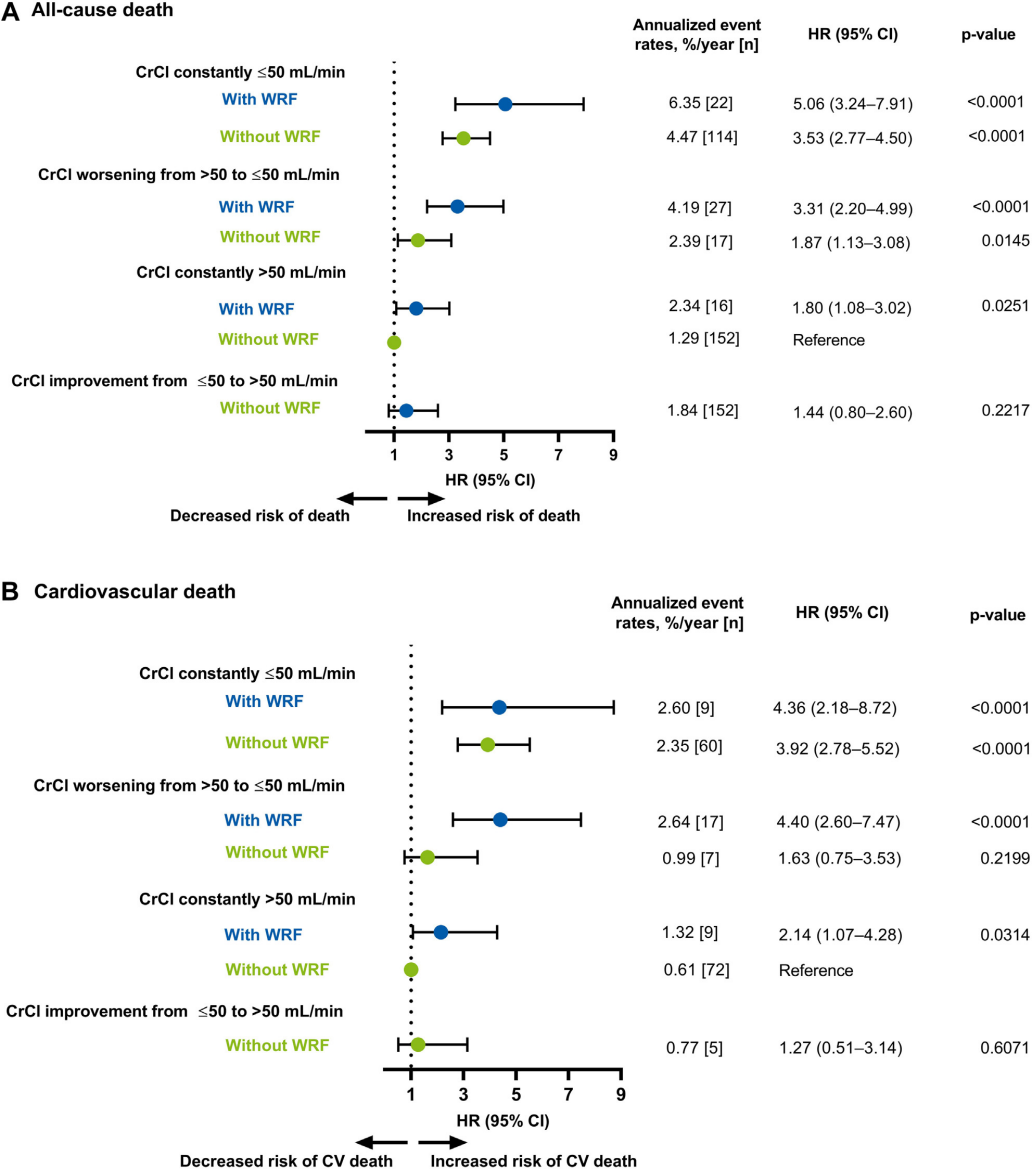

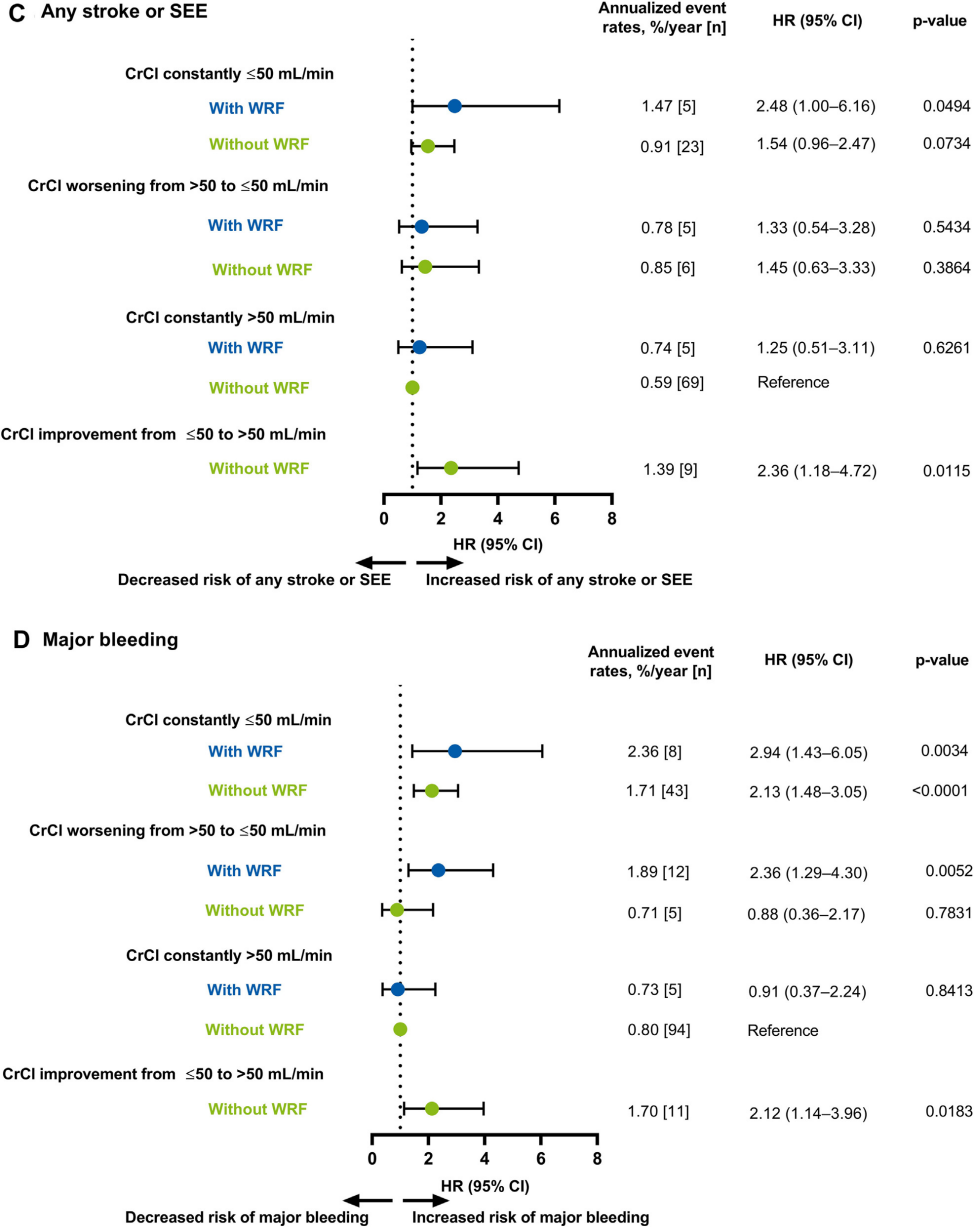

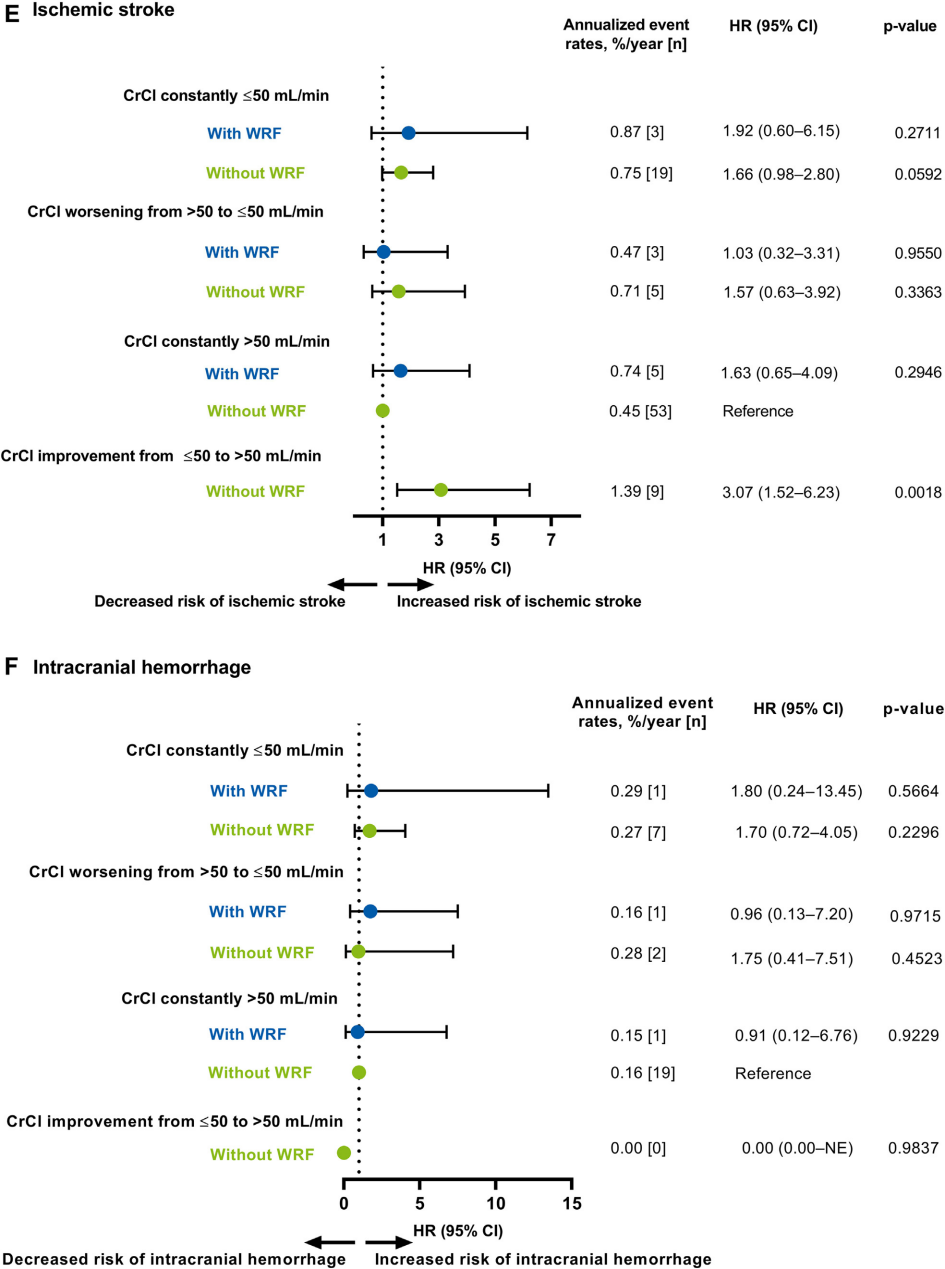
Unadjusted Association Between Worsening Renal Function and Clinical Outcomes by Creatinine Clearance Categories Forest plots illustrating clinical outcomes (A) All-cause death, (B) CV death, (C) Any stroke/SEE, (D) Major bleeding, (E) Ischemic stroke, and (F) Intracranial hemorrhage in patients categorized by CrCl. Data are represented as annualized event rates, %/y (n); ‘CrCl constantly >50 mL/min without WRF’ was used as the reference comparator for all categories. CI = confidence interval; CrCl = creatinine clearance; HR = hazard ratio; NE = not estimated; SEE = systemic embolic event; WRF = worsening renal function.

The risk of all-cause death was significantly higher in all CrCl categories (except the category presenting with CrCl improvement from ≤50 to >50 mL/min without WRF) compared with the reference group (ie, CrCl constantly >50 mL/min without WRF) (Figure 2A). The risk of CV death was significantly higher in all CrCl categories (except for the categories presenting with CrCl worsening from >50 to ≤50 without WRF and CrCl improvement from ≤50 to >50 mL/min without WRF) compared with the reference group (Figure 2B).

The risk of any stroke or SEE was low but was significantly higher in patients with WRF and CrCl constantly ≤50 mL/min and those with CrCl improvement from ≤50 to >50 mL/min without WRF compared with the reference group (Figure 2C). The risk of major bleeding was significantly higher in all CrCl categories (except in the categories presenting with CrCl worsening from >50 to ≤50 without WRF and CrCl constantly >50 mL/min with WRF) compared with the reference group (Figure 2D). The risk of ischemic stroke was low in all CrCl categories except significant increase noted in those with CrCl improvement from ≤50 to >50 mL/min without WRF compared with the reference group (Figure 2E). The risk of ICH was very low in all CrCl categories compared with the reference group (Figure 2F).

### Predictors of WRF and predictors of clinical outcomes

**Figure 3:**
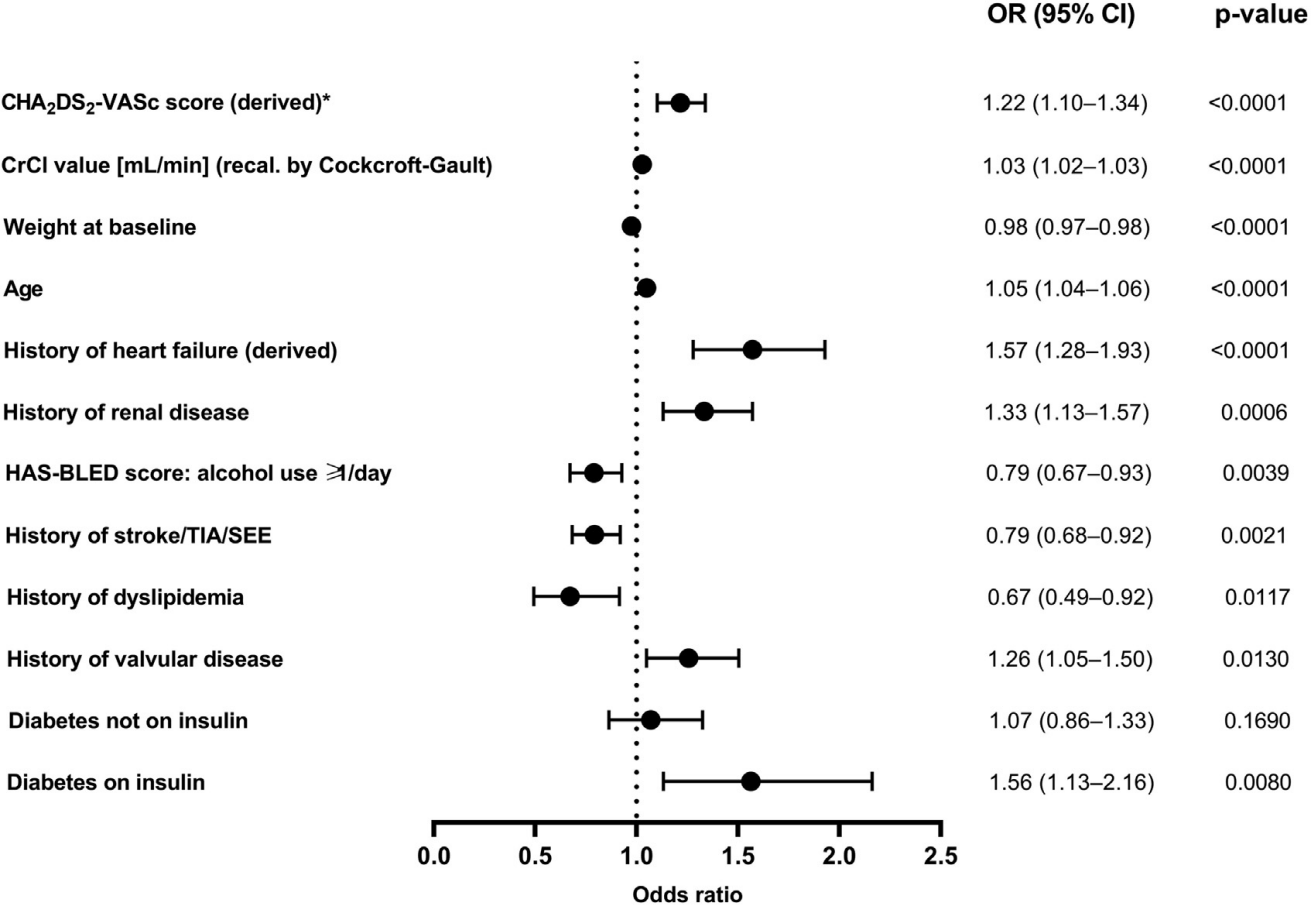
**Predictors of Worsening Renal Function** Forest plot showing predictors of WRF, identified by multivariable logistic regression model stepwise selection. Data are represented as ORs with 95% Wald Confidence Limits. ^a^CHA_2_DS_2_-VASc score excludes complex vascular plaque and was based on derived HF. A patient was considered as having a medical history of HF if 1 of the following criteria was fulfilled: documented congestive heart failure (CHF), or, if CHF was not documented, then documentation of ischemic cardiomyopathy, ejection fraction <40%, frequent dyspnea (≥1/d) without chronic obstructive pulmonary disease and with documented severe valvular heart disease, coronary heart disease post myocardial infarction, valve replacement, or hypertension treated with ≥3 drugs. CI = confidence interval; CrCl = creatinine clearance; DM = diabetes mellitus; HF = heart failure; OR = odds ratio; SEE = systemic embolic event; TIA = transient ischemic attack; WRF = worsening renal function.

The strongest predictor for WRF was high CHA_2_DS_2_-VASc score, followed by high CrCl (recalculated by Cockcroft-Gault) at baseline, low body weight, and older age (Figure 3). Predictors with an inverse association with WRF were a history of dyslipidemia, a history of stroke/transient ischemic attack (TIA)/SEE, and alcohol use ≥1 day (from HAS-BLED score) at baseline (Figure 3).

**Figure 4:**
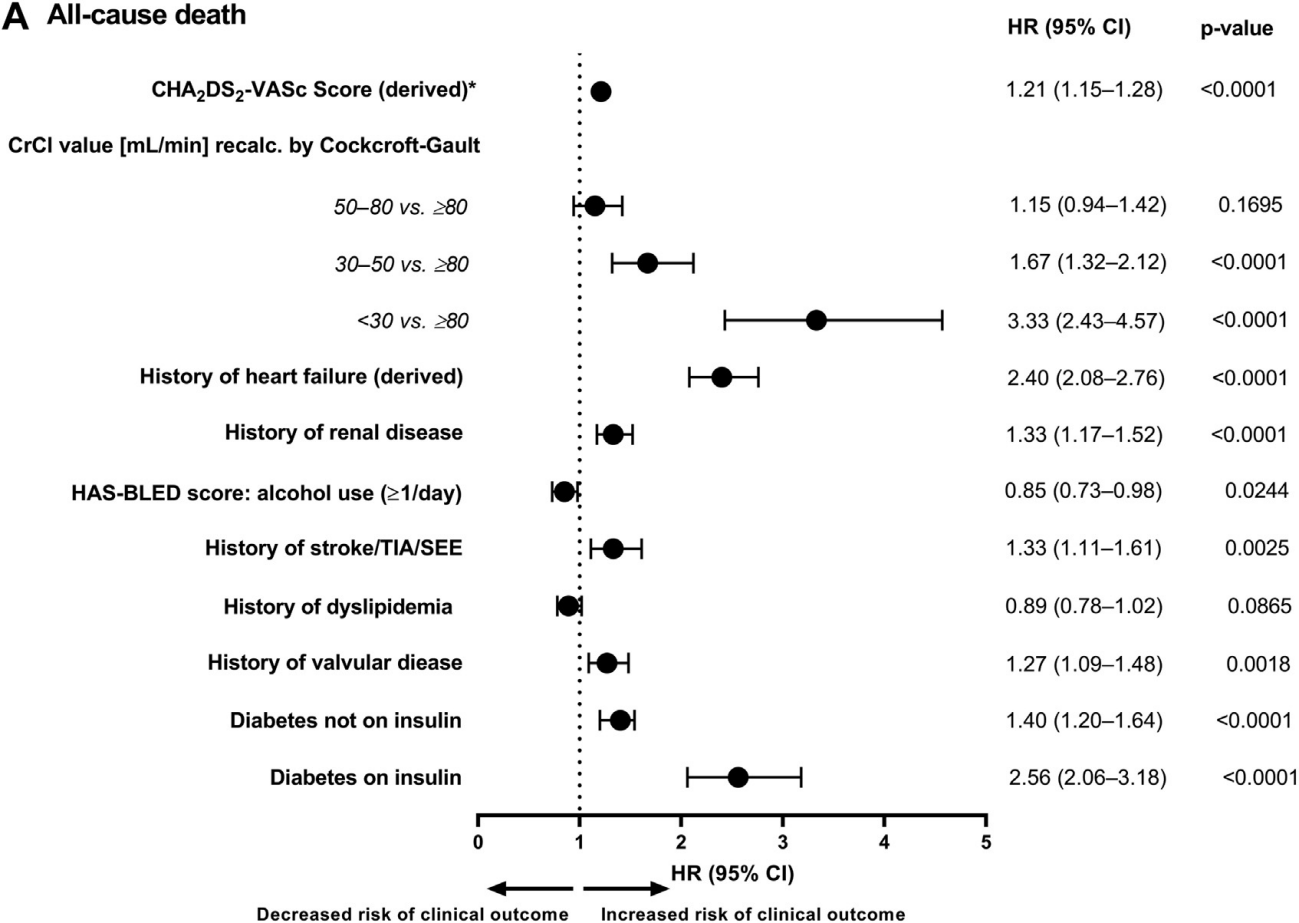

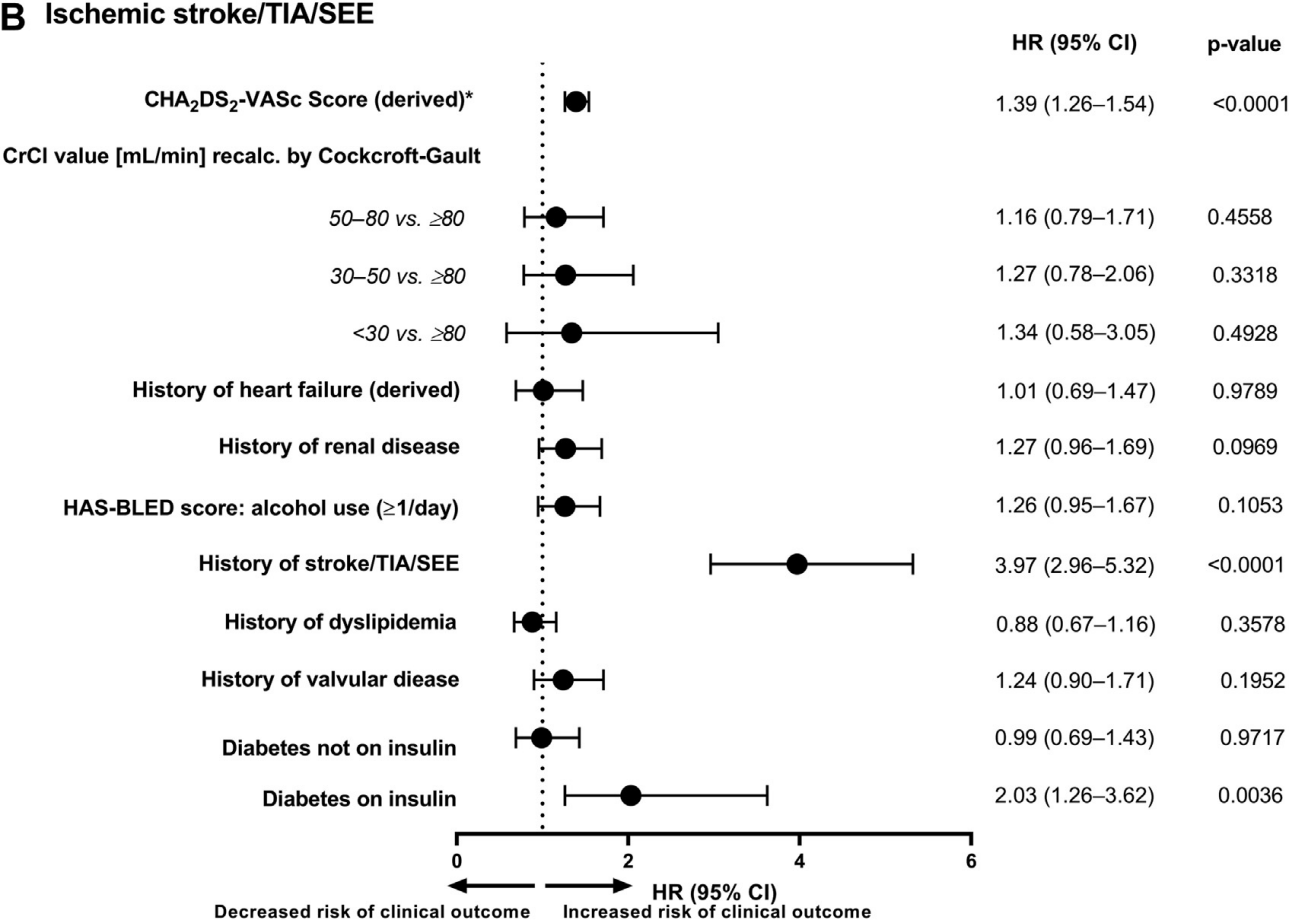

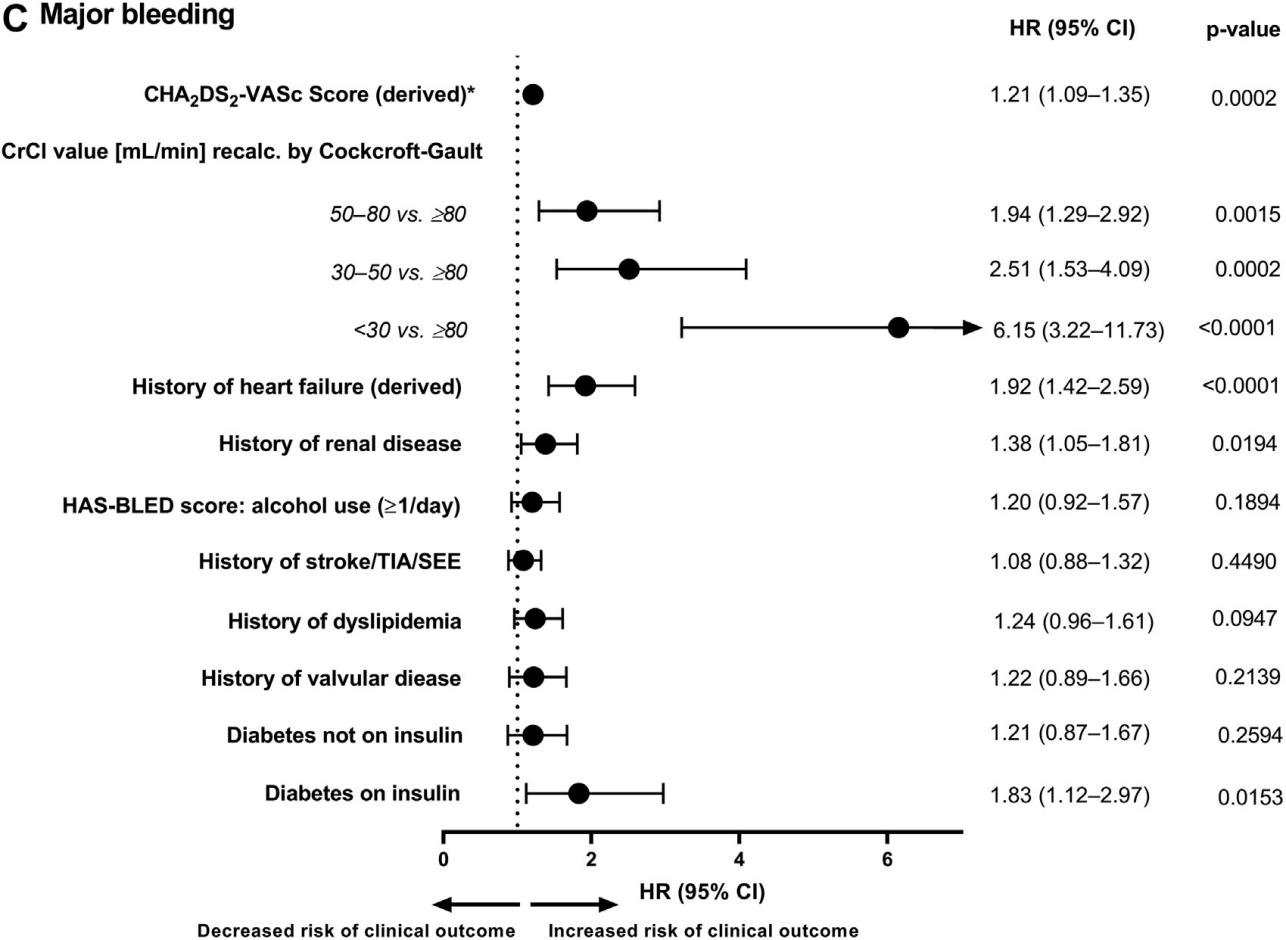
**Age-Adjusted Predictors of Adverse Clinical Outcomes** Forest plots showing age-adjusted predictors of (A) All-cause death, (B) Ischemic stroke/TIA/SEE and (C) Major bleeding, calculated using age-adjusted Cox model. Data are represented as HRs with 95% CI. ^a^CHA_2_DS_2_-VASc score excludes complex vascular plaque and was based on derived HF. A patient was considered as having a medical history of HF if 1 of the following criteria was fulfilled: documented congestive heart failure (CHF), or, if CHF was not documented, then documentation of ischemic cardiomyopathy, ejection fraction <40%, frequent dyspnea (≥1/d) without chronic obstructive pulmonary disease and with documented severe valvular heart disease, coronary heart disease post myocardial infarction, valve replacement, or hypertension treated with ≥3 drugs. CI = confidence interval; CrCl = creatinine clearance; CG = Cockcroft Gault; DM = diabetes mellitus; HF = heart Failure; HR = hazard ratio; SEE = systemic embolic event; TIA = transient ischemic attack.

Most of the predictors for WRF were also found to be predictors of clinical outcome events (Figure 4A-C). Age was a statistically significant predictor of all-cause death (HR: 1.084; 95% CI: 1.075-1.093), ischemic stroke/TIA/SEE (HR: 1.051; 95% CI: 1.034-1.068), and major bleeding (HR: 1.047; 95% CI: 1.031-1.064; *P* < 0.0001 for all 3 clinical outcomes). After adjusting for age, high CHA_2_DS_2_-VASc score, low CrCl value at baseline, history of HF, history of renal disease, history of stroke/TIA/SEE, history of valvular disease, and diabetes (mainly treated with insulin) were predictors of all-cause death (Figure 4A). Age-adjusted predictors of ischemic stroke/TIA/SEE were CHA_2_DS_2_-VASc score, history of stroke/TIA/SEE, and treatment with insulin for DM (vs no DM or DM without the need for insulin) (Figure 4B). After adjusting for age, CHA_2_DS_2_-VASc score, low CrCl value at baseline, history of HF, history of renal disease, and diabetes (mainly treated with insulin; effect of DM not treated with insulin was nonsignificant) were predictors of major bleeding (Figure 4C).

### Dosing adjustments

In the subgroup of patients with a CrCl >50 mL/min at baseline and CrCl ≤50 mL/min at 2-year follow-up, that is, 7.9% of the whole study data, 48.0% developed WRF. In contrast, in those with a CrCl constantly ≤50 mL/min at baseline and at 2-year follow-up, that is, 17.0% of the whole study data, the incidence of WRF was 12.1% (Table 2). A smaller subset of 344 patients (ie, 3.8% of the whole study data) had reduced renal function at baseline which improved over the 2-year follow-up period. In all of these subgroups, the dose of edoxaban was adjusted during the 2-year follow-up as per the label recommendations only in a minor proportion of patients (Table 2).

**Table 2:** Dosing Adjustments in Edoxaban-Treated Patients With Atrial Fibrillation During a 2-Year Follow-Up Period

Edoxaban-Treated Patients With AF (n = 9,054)				
N (%)	Worsening of Renal Function With CrCl >50 mL/min at Baseline and CrCl ≤50 mL/min at 2-y Follow-Up N = 713 (7.9%)	CrCl Constantly ≤50 mL/min at Baseline and at 2-y Follow-Up N = 1,537 (17.0%)	CrCl ≤50 mL/min at Baseline and CrCl >50 mL/min at 2-y Follow-Up N = 344 (3.8%)	CrCl Constantly >50 mL/min at Baseline and at 2-y Follow-Up N = 6,460 (71.3%)
7 Subgroups Scenario	CrCl Worsening From >50 to ≤50 mL/min With WRF [n = 342 (3.8%)] AND CrCl Worsening From >50 to ≤50 mL/min Without WRF [n = 371 (4.1%)] Together	CrCl Constantly ≤50 mL/min With WRF [n = 186 (2.1%)] AND CrCl Constantly ≤50 mL/min Without WRF [n = 1,351 (14.9%)] Together	CrCl Improvement From ≤50 to >50 mL/min [n = 344 (3.8%)]	CrCl Constantly >50 mL/min With WRF [n = 352 (3.9%)] AND CrCl Constantly >50 mL/min Without WRF [n = 6,108 (67.5%)] Together
Label recommended edoxaban 60 mg OD	447 (62.7% of 713)	412 (26.8% of 1,537)	141 (41.0% of 344)	5,777 (89.4% of 6,460)
Switched to edoxaban 30 mg OD	31 (6.9% of 447)	37 (9.0% of 412)	4 (2.8% of 141)	63 (1.1% of 5,777)
Withdrew from study drug	93 (20.8% of 447)	78 (18.9% of 412)	22 (15.6% of 141)	750 (13.0% of 5,777)
Study drug suspended	86 (19.2% of 447)	68 (16.5% of 412)	23 (16.3% of 141)	968 (16.8% of 5,777)
No documented action regarding edoxaban^a^	237 (53.0% of 447)	229 (55.6% of 412)	92 (65.2% of 141)	3,996 (69.2% of 5,777)
Edoxaban 30 mg^b^ OD	266 (37.3% of 713)	1,125 (73.2% of 1,537)	203 (59.0% of 344)	683 (10.6% of 6,460)
Switched to edoxaban 60 mg OD	2 (0.8% of 266)	6 (0.5% of 1,125)	3 (1.5% of 203)	19 (2.8% of 683)
Withdrew from study drug	46 (17.3% of 266)	275 (24.4% of 1,125)	37 (18.2% of 203)	119 (17.4% of 683)
Study drug suspended	32 (12.0% of 266)	141 (12.5% of 1,125)	31 (15.3% of 203)	81 (11.9% of 683)
No documented action regarding edoxaban^c^	186 (69.9% of 266)	703 (64.5% of 1,125)	132 (65.0% of 203)	464 (67.9% of 683)

AF = atrial fibrillation; CrCl = creatinine clearance; OD = once daily; WRF = worsening renal function.

a Most patients were treated with label recommended edoxaban 60 mg dose with no documented action regarding switching to another dose, withdrawal or suspension (apart from some missing data which were difficult to distinguish).

b Edoxaban 30 mg OD is the recommended dose in patients with 1 or more of the following clinical factors: i) moderate or severe renal impairment (creatinine clearance (CrCl) 15-50 mL/min), ii) low body weight ≤60 kg, or iii) concomitant use of the following P-glycoprotein (P-gp) inhibitors: ciclosporin, dronedarone, erythromycin, or ketoconazole.

c Most patients were treated with label recommended edoxaban 30 mg dose with no documented action regarding switching to another dose, withdrawal or suspension (apart from some missing data which were difficult to distinguish).

## Discussion

Our results show that in the majority of patients renal function remained stable and did not decline during the 2-year follow-up period. The proportion of edoxaban-treated patients with AF who experienced WRF (9.7%) is similar to the incidence rates reported in studies of patients receiving other NOACs (dabigatran, rivaroxaban, and apixaban) and lower than that reported for VKAs.^12^ Results from a meta-analysis showed the incidence rate of WRF in patients treated with NOACs (dabigatran, rivaroxaban, and apixaban) was 11.0%/y compared with 15.8%/y in the warfarin group,^12^ with the hypothesis that this was due to accelerated WRF in VKA-treated patients.^13^

In ETNA-AF-Europe, patients with WRF were older, had lower body weights, were more often frail and more likely to have underlying CV comorbidities than those without WRF. The annualized event rates for all-cause death, CV death, and major bleeding were significantly higher in patients with WRF than in those without (Central Illustration). Furthermore, and in keeping with findings for edoxaban from the Effective Anticoagulation with Factor Xa Next Generation in Atrial Fibrillation-Thrombolysis in Myocardial Infarction 48 (ENGAGE AF-TIMI 48) trial, the rates of any stroke/SEE and ischemic stroke were low in patients with and without WRF.^14^ Importantly, rates of ICH were very low in both subgroups, which is also in keeping with the findings from ENGAGE AF-TIMI 48 across different subgroups of renal function decline,^7^^,^^14^ suggesting that the risk of ICH was low in patients receiving edoxaban irrespective of renal function decline.

**Central Illustration:**
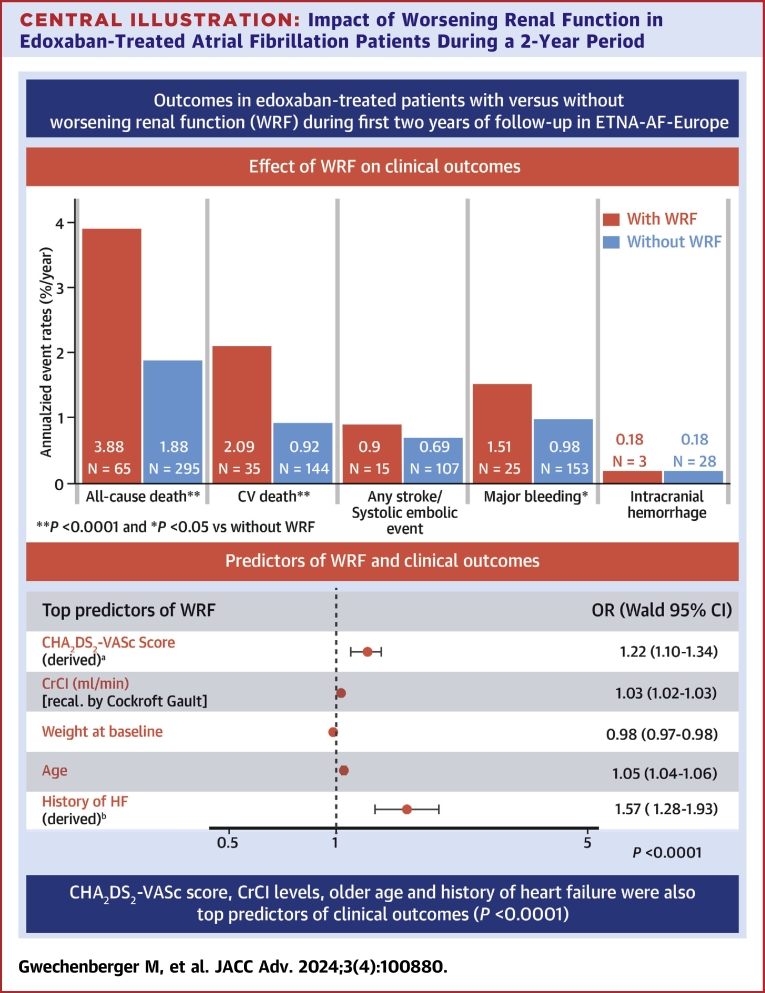
**Impact of Worsening Renal Function in Edoxaban-Treated Atrial Fibrillation Patients During a 2-Year Period** The incidence of WRF in edoxaban-treated AF patients during a 2-year period was approximately 10%. Annualized event rates of all-cause death, CV death and major bleeding were higher for patients with vs without WRF. Intracranial hemorrhage rates were low in both subgroups. Annualized rates of any stroke/SEE and ischemic stroke were low in patients with WRF, but numerically higher compared with those without WRF. The strongest predictor of WRF was high CHA_2_DS_2_-VASc score, followed by high CrCl (recalculated by Cockcroft-Gault) at baseline, low body weight, and older age, and most of the predictors for WRF were also observed to be the predictors of clinical outcomes. ^a^CHA2DS2-VASc score excludes complex vascular plaque and was based on derived HF. ^b^A patient was considered as having a medical history of HF if 1 of the following criteria was fulfilled: documented congestive heart failure (CHF), or, if CHF was not documented, then documentation of ischemic cardiomyopathy, ejection fraction <40%, frequent dyspnea (≥1/d) without chronic obstructive pulmonary disease and with documented severe valvular heart disease, coronary heart disease post myocardial infarction, valve replacement, or hypertension treated with ≥3 drugs. AF = atrial fibrillation; CAD = coronary artery disease; CHA2DS2-VASc = congestive heart failure, hypertension, age 75 years or older, diabetes mellitus, stroke; COPD = chronic obstructive pulmonary disease; CrCl = creatinine clearance; CV = cardiovascular; ICH = intracranial hemorrhage; LVEF = left ventricular ejection fraction; SEE = systemic embolic event; WRF = worsening renal function.

Overall, the additional categorization analyses suggest that patients with and without WRF were ∼2 to 5 times more likely to die due to any cause in categories of CrCl constantly ≤50 mL/min, CrCl worsening from >50 to ≤50 mL/min, and CrCl constantly >50 mL/min with WRF compared with the reference group (ie, CrCl >50 mL/min during 2 years of follow-up without WRF). Similarly, patients with and without WRF were ∼2 to 4 times more likely to die from CV death in CrCl constantly ≤50 mL/min, CrCl worsening from >50 to ≤50 mL/min, and CrCl constantly >50 mL/min with WRF categories compared with the reference group. This further illustrates that patients with declining renal function are at an increased risk of death or a clinical outcome event.

Only patients with WRF and CrCl constantly ≤50 mL/min and patients with CrCl improvement from ≤50 to >50 mL/min were ∼2 times more likely to have any stroke or SEE compared with the reference group. The higher incidence of any stroke or SEE in patients with CrCl improvement from ≤50 to >50 mL/min is apparently an odd finding without reasonable explanation and may have been a chance finding due to the low numbers in this group. The risk of major bleeding was approximately 2 to 3 fold higher in patients with and without WRF in CrCl constantly ≤50 mL/min and in patient with WRF in CrCl worsening from >50 to ≤50 mL/min categories, compared with the reference group.

Most patients in this study were given the label-recommended dose of edoxaban at baseline. However, of those patients whose renal function worsened or improved during the 2-year follow-up period, the dose of edoxaban was adjusted accordingly only in a minor proportion of patients. Notably, withdrawal or suspension of edoxaban administration was apparently more common than dose adjustments, which may suggest a gap and the need in clinical practice to more closely monitor renal function and adjust the dosage of NOACs according to the label recommendations in order to optimize the management of patients with AF and renal decline. Indeed, there is a lack of evidence-based recommendations for the management of patients with AF and declining renal function, especially in those with severe CKD or end-stage renal disease on dialysis. Together with conflicting findings from observational cohort studies, the ongoing challenges in the management of these vulnerable patients who require dose adjustments still remain.^15^

### Predictors of WRF and clinical outcomes

CHA_2_DS_2_-VASc score, high baseline CrCl levels, low body weight, older age, and history of HF, renal disease, valvular disease, and diabetes were identified as the main predictors of WRF (Central Illustration). It is not surprising that the CHA_2_DS_2_-VASc score was the strongest predictor of WRF because it includes several variables individually related to WRF. Neither is the finding that only diabetes (on insulin treatment) was correlated with WRF, despite the fact that many more diabetic patients not on insulin were in this subgroup analysis, because earlier reports by Patti et al (2017)^16^ demonstrated a significantly higher risk of stroke/systemic embolism in insulin-treated diabetes patients vs no diabetes or diabetes without insulin treatment. Moreover, although high baseline CrCl was unsurprisingly a strong predictor of WRF, it is of importance to note that a 25% decline in renal function in these patients with high baseline levels of CrCl are unlikely to be of clinical concern (Table 3).

**Table 3:** Creatinine Clearance and Related Parameters

Variable	N	N miss	Mean (SD)	Minimum	Fifth percentile	Median (IQR)	95^th^ Percentile	Maximum
Baseline								
CrCl (recalculated by Cockcroft-Gault)	11,440	1,693	74.3 (30.4)	13.3	34.4	69.8 (53.0-89.6)	129.6	315.2
Serum creatinine^a^ [mg/dL]	11,756	1,377	1.0 (0.3)	0.3	0.6	1.0 (0.8-1.1)	1.6	7.7
2-y follow-up with last observation carried forward								
CrCl (recalculated by Cockcroft-Gault)	9,495	3,638	71.7 (30.5)	7.2	31.7	67.1 (50.4-87.4)	128.1	272.1
Ratio CrCl 2 y/baseline	9,054	4,079	1.0 (0.2)	0.1	0.7	1.0 (0.9-1.1)	1.3	2.0
Serum creatinine^a^ [mg/dL]	9,750	3,383	1.1 (0.4)	0.3	0.7	1.0 (0.8-1.2)	1.7	10.3
CrCl at 2-y follow-up without last observation carried forward								
CrCl (recalculated by Cockcroft-Gault)	7,374	5,759	71.6 (30.0)	8.5	32.5	67.0 (50.6-87.0)	127.5	272.1
Ratio CrCl 2 y/baseline	7,089	6,044	1.0 (0.2)	0.1	0.7	1.0 (0.9-1.1)	1.3	2.0
Serum creatinine [mg/dL]	7,560	5,573	1.1 (0.4)	0.3	0.7	1.0 (0.8-1.2)	1.7	10.3

CrCl = creatinine clearance; IQR = interquartile range; SD = standard deviation.

a Serum creatinine numbers are slightly higher than that for CrCl (recalculated by Cockcroft-Gault) owing to missing body weight data.

Contrarily, history of dyslipidemia was associated with a reduced risk of WRF; this may be due to the fact that in the majority of patients statins were used, which could provide a protective effect against WRF.^17^ Indeed it has been previously reported that statin-treated patients were associated with slightly less worsening of renal function over time, although that may have been due to the degree of progression of kidney disease.^18^ Moreover, available data suggest that statins do not only preserve but also increase renal function.^19^ As in ETNA-AF-Europe, there are no data on lipid profiles,^6^ but only on medication use, this possible explanation for the lower risk of WRF remains speculative.

History of stroke/TIA/SEE also appeared to have been an inverse predictor of WRF; but this lower incidence is merely a consequence of the decision to include predictor variables in the multivariable model that explain the same variance in WRF among the cohort, that is, the effect of previous stroke/TIA/SEE as a predictor of WRF is weaker because it is already a component of the CHA_2_DS_2_-VASc score (which is the strongest predictor of WRF). Additionally, previous HF (also shown to be a predictor of WRF) and history of stroke/TIA/SEE are correlated factors, and this further adds to weakening the hypothesis of previous stroke/TIA/SEE as an independent predictor of WRF leading to an inverse association.

Most of the WRF predictors such as CHA_2_DS_2_-VASc score, CrCl levels, history of HF, history of renal disease, and history of stroke/TIA/SEE were also predictors for clinical outcome events (Central Illustration). Advancing age is an important predictor of WRF and as it was the most prominent risk factor for AF-related outcomes, the predictors of clinical outcomes were adjusted for age in order to assess the residual effect of the other risk factors on the various clinical outcomes.

### Limitations of this subanalysis

The major limitations to this substudy are that: (A) our hypotheses were defined post hoc, (B) a substantial proportion of patients were excluded from our analyses because the serum creatinine assessment was not done; (C) our dataset only had 1 creatinine measurement per visit, whereas in clinical practice it is possible that repeat measurements are taken to ascertain if a dose adjustment is indicated, especially in patients with suspected acute causes of renal decline; and (D) we had to fill-in some of the missing renal function data using the last observation carried forward approach. A consequence of this imputation method is that all patients who had not developed WRF prior to the missing renal function data time point were presumed and thus classified as not having developed WRF thereafter. In addition, other factors, for example, acute diseases, renal decline due to nephrotoxic contrast agents, or drugs/medication received during hospitalization for HF that may cause WRF during this period are not taken into account. Furthermore, there are at least 4 different definitions for WRF in the literature (a >20% decrease in CrCl;^20^ a decrease in estimated glomerular filtration rate (GFR) by 20 to 30%;^9^^,^^12^^,^^21^ an increase in serum creatinine by >0.3 mg/dL;^12^ and doubling of serum creatinine).^12^ A decrease in CrCl by ≥25% from baseline was used here as the definition for WRF, in line with the definitions used in several papers in the literature. However, in other NOAC studies, different definitions for WRF have been used, which makes comparisons between NOACs challenging. The definition used in Rivaroxaban Once daily oral direct actor Xa inhibition Compared with vitamin K antagonism for prevention of stroke and Embolism Trial in Atrial Fibrillation (ROCKET-AF) trial was >20% decrease from screening CrCl at any time during the study period; in the Apixaban for Reduction in Stroke and Other Thromboembolic Events in Atrial Fibrillation (ARISTOTLE) trial, annual decrease in estimated GFR ≥20% was used; whereas in RE-LY a decrease in GFR >25% from baseline was used.^9^^,^^20^^,^^21^ Because of our definition of WRF, we cannot ascertain if patients developed WRF before or after the outcome events. In addition, some of the findings for the predictors of WRF are physiologically less plausible, for example, high CrCl at baseline, and were possibly due to the definition of WRF used, the prevalence of high baseline CrCl values in the majority of patients, or both. The inability to ascertain timing of outcome events in relation to WRF is also an important study limitation and can be further impacted by the high number of missing creatinine samples. Moreover, thromboembolic event rates were low in this post hoc analysis, with wide CIs in i) ICH and ii) patients with improved CrCl for ischemic stroke rates. Thus, raising the question regarding the power to detect any such difference.

Another study limitation is that the risk model used in this subanalysis is yet to be validated by an independent cohort and the bootstrapping results are not presented in this paper, denoting the fully exploratory nature of the results. Although it is not uncommon for such validation studies to be published separately; the aforementioned inherent limitations of this type of study should be duly noted. Importantly, the potential predictors were chosen from all of the data collected by clinical experts; therefore, they make clinical sense. However, in the absence of a validation study, the conclusions regarding risk models appear weakened and emphasize the need for validating the model by an independent cohort.

## Conclusions

The incidence of WRF in AF patients treated with edoxaban over a 2-year period was approximately 10%, similar to that reported for other NOACs. The rates of death and major bleeding were significantly higher in patients with WRF than in those without such decline in renal function. While WRF is associated with a higher risk of death, it is not associated with stroke in this cohort. Annualized rates of any stroke/SEE were low in patients with WRF, but higher compared to those without WRF, and importantly, ICH rates were very low irrespective of WRF. The strongest predictors for WRF were high CHA_2_DS_2_-VASc score, high CrCl, low body weight, and older age; most of which were also found to be predictors of clinical outcome events. Overall, these results support findings from randomized clinical trials of edoxaban regarding protection against strokes and bleeding events, irrespective of the presence of WRF.

PERSPECTIVES **COMPETENCY IN MEDICAL KNOWLEDGE 1:** Renal function is a dose reduction criterion for NOACs. It is key to consider how patients with AF whose renal function worsens over time should be treated.

**COMPETENCY IN MEDICAL KNOWLEDGE 2:** The incidence of WRF in edoxaban-treated patients with AF is approximately 10%. Indirect comparisons with other studies indicate that the real-world evidence from this study supports findings from randomized trials of edoxaban in terms of protection against strokes and bleeding events.

**TRANSLATIONAL OUTLOOK 1:** Withdrawal or suspension of edoxaban administration was seemingly more common than dose adjustments in this study, suggesting the need to more closely monitor renal function and adjust the dosage of NOACs as per summary of product characteristics recommendations in order to better manage patients with AF and renal decline, and to prevent discontinuation of anticoagulation in high-risk patients with AF.

**TRANSLATIONAL OUTLOOK 2:** Additional research is needed to understand the safety and efficacy of NOACs in patients with AF and declining renal function, such as those with severe CKD or end-stage renal disease on dialysis.

## Funding support and author disclosures

This study was funded by 10.13039/501100022274Daiichi Sankyo Europe GmbH, Munich, Germany. Dr Gwechenberger has received personal fees (lectures, advisory boards, research grants) and travel grants from Daiichi Sankyo, Boehringer Ingelheim, Bayer, Abbott, Biotronik, Boston, Medtronic, and Sorin. Dr Barón-Esquivias has received honoraria for presentations and/or consultancy fees and/or research grants from Boehringer Ingelheim, Bayer, Daiichi Sankyo, Pfizer-Bristol-Myers-Squibb, and Biotronik. Dr de Vries has received nonfinancial support from Daiichi Sankyo for other research projects related to the ETNA-AF-Europe registry, and speaker fees from Bristol Myers Squibb. He is also being considered for the adjudication committee of the LIMIT & DANCE trials, which are sponsored by the Population Health Research Institute (PHRI). Dr Siller-Matula has received speaker or consultant fees from Chiesi, Biosensors, Boston Scientific, P&F, Boehringer Ingelheim, and Daiichi Sankyo not related to the submitted work. Dr de Groot reports personal fees from Daiichi Sankyo during the conduct of the study; grants from Abbott, Atricure, Bayer, Boston Scientific, Daiichi Sankyo, Johnson & Johnson, and Medtronic; personal fees from Atricure, Bayer, berlin-Chemie, Daiichi Sankyo, Johnson & Johnson, Medtronic, Menarini, Novartis, and Servier; and other from RhythmCARE outside the submitted work. Dr Manu was an employee of Daiichi Sankyo Europe GmbH, Munich, Germany (at the time of development of the manuscript). Dr Souza is an employee of Daiichi Sankyo Europe GmbH, Munich, Germany. Dr Wienerroither is an employee of Daiichi Sankyo Austria GmbH, Vienna, Austria. Dr Pecen has received fees and honoraria from Daiichi-Sankyo, SOTIO, and Beckman Coulter. Dr De Caterina reports grants, personal fees and nonfinancial support from Daiichi Sankyo, during the conduct of the study; and reports consulting fees, honoraria and research funding from: AstraZeneca, Boehringer Ingelheim, Bayer, BMS/Pfizer, Daiichi Sankyo, Janssen, Milestone, Novartis, Merck, Portola, Sanofi, Menarini, Guidotti, and Roche, outside the submitted work. Dr Kirchhof receives research support for basic, translational, and clinical research projects from European Union Big-Data@Heart (grant agreement EU IMI 116 074); CATCH ME (grant agreement ID: 633 196); AFFECT-EU (grant agreement ID: 847 770); Leducq foundation, Medical Research Council (UK); German Centre for Cardiovascular Research supported by the German Ministry of Education and Research; from several drug and device companies active in atrial fibrillation and has received honoraria from several such companies in the past but not in the last 3 years. He is listed as inventor on 2 patents held by University of Birmingham (Atrial Fibrillation Therapy WO 2 015 140 571, Markers for Atrial Fibrillation WO 2 016 012 783) and is employed as Director of the Department of Cardiology, University Heart and Vascular Centre UKE Hamburg and Professor of Cardiovascular Medicine (part-time), University of Birmingham, UK. He is also Speaker of the board of AFNET, Germany, and Board member of the ESC.
