# Supplementary material for: Incidence and Predictors of Worsening Renal Function in Edoxaban-Treated Atrial Fibrillation Patients Within ETNA-AF-Europe Registry
Source: JACC Adv. 2024 Mar 11;3(4):100880. doi: 10.1016/j.jacadv.2024.100880 (PMC11198551; doi:10.1016/j.jacadv.2024.100880)
Supplement: Appendix [file mmc1.pdf]

Supplemental table

Supplemental Table 1: Baseline characteristics of edoxaban-treated patients with AF categorized by CrCl levels during a 2-year follow-up period.

	**CrCl constantly ≤50 mL/min with WRF**	**CrCl constantly ≤50 mL/min without WRF**	**CrCl worsening from >50 to ≤50 mL/min with WRF**	**CrCl worsening from >50 to ≤50 mL/min without WRF**	**CrCl constantly >50 mL/min with WRF**	**CrCl constantly >50 mL/min without WRF**	**CrCl improvement from ≤50 to >50 mL/min**	**p-value**
	**[n=186 (2.1%)]**	**[n=1351 (14.9%)]**	**[n=342 (3.8%)]**	**[n=371 (4.1%)]**	**[n=352 (3.9%)]**	**[n=6108 (67.5%)]**	**[n=344 (3.8%)]**	
Edoxaban dose at baseline								
60 mg, OD	45 (24.2)	367 (27.2)	252 (73.7)	253 (68.2)	327 (92.9)	5450 (89.2)	141 (41.0)	<0.0001
30 mg, OD	141 (75.8)	984 (72.8)	90 (26.3)	118 (31.8)	25 (7.1)	658 (10.8)	203 (59.0)	<0.0001
Overall adherence to SmPC								
Recommended edoxaban dose at baseline	141 (75.8)	984 (72.8)	261 (76.3)	252 (67.9)	320 (90.9)	5378 (88.0)	203 (59.0)	<0.0001
Non-recommended edoxaban dose at baseline	45 (24.2)	367 (27.2)	81 (23.7)	119 (32.1)	32 (9.1)	730 (12.0)	141 (41.0)	<0.0001
Male	72 (38.7)	488 (36.1)	161 (47.1)	169 (45.6)	194 (55.1)	3850 (63.0)	157 (45.6)	<0.0001
Age [years]	82.6 ± 5.8	82.3 ± 6.1	77.4 ± 7.5	79.2 ± 5.5	70.6 ± 9.0	71.1 ± 8.8	79.1 ± 6.8	<0.0001
Weight [kg]	68.9 ± 12.3	67.3 ± 12.1	75.5 ± 14.3	72.6 ± 11.9	86.6 ± 18.4	84.8 ± 16.7	72.7 ± 12.3	<0.0001
Body mass index [kg/m^2]	25.9 ± 3.9	25.2 ± 4.0	27.4 ± 4.8	26.2 ± 3.8	29.8 ± 5.7	28.9 ± 5.1	26.5 ± 4.2	<0.0001
Recalc. CrCl[a] (CG formula) [mL/min] baseline	39.8 ± 7.4	38.6 ± 7.5	66.1 ± 13.0	55.2 ± 3.9	104.7±31.4	84.5 ± 26.3	44.7 ± 4.5	<0.0001
≥ 80	0 (0.0)	0 (0.0)	45 (13.2)	0 (0.0)	294 (83.5)	2900 (47.5)	0 (0.0)	<0.0001
50; 80	0 (0.0)	0 (0.0)	297 (86.8)	371 (100.0)	58 (16.5)	3208 (52.5)	0 (0.0)	<0.0001
30; 50	162 (87.1)	1157 (85.6)	0 (0.0)	0 (0.0)	0 (0.0)	0 (0.0)	344 (100.0)	<0.0001
15; 30	24 (12.9)	193 (14.3)	0 (0.0)	0 (0.0)	0 (0.0)	0 (0.0)	0 (0.0)	<0.0001

<15	0 (0.0)	1 (0.1)	0 (0.0)	0 (0.0)	0 (0.0)	0 (0.0)	0 (0.0)	<0.0001
Recalc. CrCl[a] (CG formula) [mL/min] 2 ys FU (LOCF)	25.6 ± 6.3	38.4 ± 7.4	38.8 ± 7.3	46.4 ± 2.7	69.7 ± 19.1	84.6 ± 27.4	57.6 ± 7.0	<0.0001
Recalc. CHA_2DS_2-VASc[b]	4.3 ± 1.2	4.2 ± 1.2	3.8 ± 1.2	3.9 ± 1.2	3.1 ± 1.4	2.9 ± 1.4	3.8 ± 1.2	<0.0001
Recalc. mod. HAS-BLED[c]	3.1 ± 1.0	3.1 ± 1.0	2.8 ± 1.0	2.9 ± 1.0	2.4 ± 1.0	2.4 ± 1.1	2.9 ± 1.0	<0.0001
Type of atrial fibrillation								
Paroxysmal	77 (41.4)	673 (49.9)	155 (45.3)	195 (52.6)	179 (51.0)	3297 (54.1)	160 (46.6)	<0.0001
Persistent	49 (26.3)	298 (22.1)	97 (28.4)	85 (22.9)	95 (27.1)	1536 (25.2)	91 (26.5)	<0.0001
Long-standing persistent	4 (2.2)	28 (2.1)	9 (2.6)	10 (2.7)	8 (2.3)	161 (2.6)	5 (1.5)	<0.0001
Permanent	56 (30.1)	350 (25.9)	81 (23.7)	81 (21.8)	69 (19.7)	1103 (18.1)	87 (25.4)	<0.0001
Perceived frailty	61 (32.8)	390 (28.9)	68 (19.9)	67 (18.1)	32 (9.1)	380 (6.2)	84 (24.4)	<0.0001
History of								
Diabetes mellitus	66 (35.5)	356 (26.4)	100 (29.2)	89 (24.0)	82 (23.3)	1309 (21.4)	68 (19.8)	<0.0001
Hypertension	163 (87.6)	1110 (82.2)	282 (82.5)	300 (80.9)	291 (82.7)	4626 (75.7)	272 (79.1)	<0.0001
Heart failure (derived)[d]	49 (26.3)	285 (21.1)	82 (24.0)	70 (18.9)	67 (19.0)	724 (11.9)	74 (21.5)	<0.0001
Peripheral artery disease	10 (5.4)	59 (4.4)	12 (3.5)	13 (3.5)	10 (2.8)	188 (3.1)	19 (5.5)	0.0445
Coronary heart disease	54 (29.0)	345 (25.5)	83 (24.3)	91 (24.5)	61 (17.3)	1216 (19.9)	86 (25.0)	<0.0001
Ischemic stroke	11 (5.9)	111 (8.2)	22 (6.4)	27 (7.3)	25 (7.1)	342 (5.6)	19 (5.5)	0.0213
Any bleeding	9 (4.8)	65 (4.8)	16 (4.7)	13 (3.5)	7 (2.0)	183 (3.0)	13 (3.8)	0.0114
Valvular disease	61 (32.8)	324 (24.0)	74 (21.6)	67 (18.1)	53 (15.1)	866 (14.2)	66 (19.2)	<0.0001

Data are presented as mean ± standard deviation for continuous variables or as number (%) for categorical variables; p-values test for statistically significant differences between any of the 7 categories. [a]CrCl was estimated by Cockcroft-Gault formula. [b]Not including complex vascular plaque, and the score was based on derived heart failure; [c]Not including labile INR, alcohol use was defined as ≥1 unit/day,and defining the presence or absence of renal or hepatic disease was left to the discretion of the physician. [d]HF was defined using the

following criteria: patients with documented congestive HF, documented ischemic cardiomyopathy, LVEF <40%, or frequent dyspnea ($\geq$ 1/day) without COPD and at least one of the following: documented severe valvular heart disease, documented CAD post myocardial infarction, valve replacement, or documented hypertension treated with at least 3 drugs. CAD, coronary artery disease; CG, Cockcroft-Gault; CHF, congestive heart failure; COPD, chronic obstructive pulmonary disease; CrCl, creatinine clearance; FU, follow-up; HF, heart failure; INR, international normalized ratio; LOCF, last observation carried forward; LVEF, left ventricular ejection fraction; OD, once daily; SmPC, summary of product characteristics; WRF, worsening renal function.

APPENDIX

List of ETNA-AF-Europe investigators

Austria

Thomas Weiss, Klinik Ottakring, Wien; Marcus Müllner, Internistisches Zentrum Brigittenau, Wien; Andreas Winkler, Klinik Pirawarth, Bad Pirawarth; Daniel Scherr, Universitätsklinik Graz, Graz; Alexander Kober, Ordination, Sankt Aegyd am Neuwalde; Peter Kaserer, Salzburg; Johann Auer, KH St. Josef Braunau, Braunau; Marianne Gwechenberger, Allgemeines Krankenhaus Medizinische Universität Wien, Wien; Johannes Foechterle, Ordination, Linz; Hans Offenbacher, LKH Knittelfeld, Knittelfeld; Norbert Watzinger, LKH Feldbach, Feldbach; Anna Rab, LKH Villach, Villach; Stephan Schoiswohl, LKH Steyr, Steyr; Petra Kleewein, Ordination, Fohnsdorf; Gerhard Puhr, KRAGES Burgenlaendische Krankenanstalten - LH Güssing, Güssing; Ventzislav Petkov, AKH - Medizinische Universität Wien, Wien; Christian Brenneis, Reha Zentrum Münster, Münster; Heinrich Weber, Kardio Weberm, Wien; Georgios Kollias, Krankenhaus der Elisabethinen Linz, Linz; Clemens Steinwender, Kepler Universitätsklinikum, Linz; Gerald Maurer, Ordinationszentrum an der Wiener Privatklinik (Ordination Group Practice Center), Wien; Heinz Drexel, VIVIT Institut at the academic teaching hospital of Feldkirch, Feldkirch; Gudrun Zweiker, Praxis Dr. Zweiker Gudrun, Straden; Hamid Schirasi-Fard, Hamid Oberrat Schirasi-Fard, Wien; Michael Gottsauner-Wolf, Office of Michael Gottsauner-Wolf, MD, Wien; Georg Röggla, Landesklinikum Neunkirchen, Neunkirchen; Alexander Erben, Praxis Dr. Höll Peter, Stainz; Reinhard Sock, Gruppenpraxis, Wien; Thomas Sycha, General University Hospital of Vienna, Vienna; Peter Siostrzonek, A. Oe. Kh Der Barmherzigen Schwestern, Linz; Milan R. Vosko, Kepler Universitätsklinikum, Linz; Jozsef Egresits, Klinikum Klagenfurt, Klagenfurt am Wörthersee; Paul Pinter, Ordination Pinter,

Leobersdorf; Robert Buder, Konventhospital der Barmherzigen Brüder Linz, Linz an der Donau; Hans Domanovits, AKH - Medizinische Universität Wien, Wien; Karl-Heinz Karner, Villach.

Belgium

Hans Vandekerckhove, AZ Sint-Lucas, Gent; Geert Hollanders, Private Practice Cardiology, De Pinte; Becker Alzand, AZ Glorieux, Ronse; Filip De Man, Europa Ziekenhuizen, Brussels; Philippe Vanduynhoven, Algemeen Stedelijk Ziekenhuis, Aalst; Frank Cools, A.Z. Klina, Brasschaat; Tom Sarens, AZ Sint-Blasius, Dendermonde; Jaak Mortelmans, Oostham; Guido Mehuys, Tielt; Rachid Maamar, Centre Hospitalier Hutois, Huy; Raf Roelandt, VZW Regionaal Ziekenhuis Jan Yperman, Leper; Luc Capiau, BVBA Dr. Luc Capiau, Wetteren; Geert Vervoort, AZ Sint-Maarten, Mechelen; Ivan Blankoff, CHU de Charleroi - Hôpital André Vésale, Montigny-le-Tilleul; Bruno Pirenne, Clinique Saint-Pierre, Ottignies; Geert Valgaeren, AZ Monica, Deurne; Marc De Tollenaere, Algemeen Ziekenhuis West, Veurne; Yvan Calozet, Gribomont; Johan Vijgen, ETC Jessa Ziekenhuis, Hasselt; Raf Brouns, Universitair Ziekenhuis Brussel, Brussels; Laetitia Yperzeele, Universitair Ziekenhuis Antwerpen, Edegem; Nina De Klippel, ETC Jessa Ziekenhuis, Hasselt; Stéphane Heijmans, Research Link, Linkebeek; Tom Rossenbacker, Imeldaziekenhuis, Bonheiden; Roeland Dierickx, Huisartsen Dierickx, Westouter; Filip Vanstechelman, St Elisabeth Ziekenhuis, Zonnegem; Axel Caenepeel, Practice Dr. Axel Caenepeel, Willebroek; Rene Tavernier, AZ Sint-Jan, Brugge; Tim Boussy, AZ Groeninge, Kortrijk; Fernand Lenoir, médecine générale, Roux; Tom De Potter, Onze Lieve Vrouw Ziekenhuis, Aalst; Emmanuel Catez, C.H.U. Brugmann - Site Victor Horta, Brussels; Joeri Voet, Vitaz, Sint-Niklaas; Carlo de Asmundis, Universitair Ziekenhuis Brussel, Brussels; Geert Huisarts, Huisartsenpraktijk Vileyn bvba, Blankenberge; Peter Goethals, Clinique St Jean, Brussels; Jos Vanhoof, Artsengroep

Zorgpoort, Lommel; Dirk Verleyen, AZ Sint-Lucas, Brugge; Etienne Hoffer, C. H. R. de la Citadelle, Liège; Frederic Marenne, St-Nikolaus Hospital, Eupen; Gaetano Paparella, Cliniques de l'Europe St-Michel, Brussels; Hendrik Celen, Regional Ziekenhuis Heilig Hart, Leuven; Maximo Rivero, Ziekenhuis Oost-Limburg, Genk; Peter Put, St-Franciskus Ziekenhuis, Heusden-Zolder; Philippe Evrard, Clinique CHC MontLégia, Liège; Frank Provenier, AZ Maria Middelares, Gent; Ruben Casado Arroyo, Cliniques Universitaires de Bruxelles Hopital Erasme, Brussels; Georges Mairesse, Cliniques du Sud-Luxembourg, Arlon; Philip-Emmanuel Baetslé, St-Dimpna Ziekenhuis, Geel; Dimitri Hemelsoet, Universitair Ziekenhuis Gent, Gent; Peter Vanacker, AZ Groeninge, Kortrijk; John Thoeng, AZ Turnhout - Campus Sint-Elisabeth, Turnhout; Thomas Vanassche, UZ Leuven, Leuven; Pierre Hausman, Grand Hôpital de Charleroi, Charleroi; Dirk Faes, Maria Ziekenhuis Noord-Limburg vzw, Overpelt; Bruno Schwagten, ZNA Middelheim, Antwerpen; Michel De Pauw, Universitair Ziekenhuis Gent, Gent; Stefan Verstraete, AZ Zeno, Knokke-Heist; Karl Dujardin, AZ Delta, Roeselare; Mehran Tahmaseb, AZ Sint-Jan, Oostende; Geert Vanhooren, AZ Sint-Jan, Brugge; Marie-Dominique Gazagnes, C.H.U. Brugmann - Site Victor Horta, Brussels; Hein Heidbuchel, Universitair Ziekenhuis Antwerpen, Edegem; Luc Piérard, CHU de Liège; Olivier Xhaet, CHU UCL Namur, Mont-Godinne, Yvoir; Stéphan Chevalier, Centre de Cardiologie Ottignies, Ottignies.

Germany

Muwafeg Abdel-Qader, Praxis Abdel-Qader, Winsen; Patrick Abdul-Malak, Gemeinschaftspraxis Dr. Patrick Abdul-Malak & Reinhard Lieb, Bahrdorf; Wolfgang Albrecht, Praxis Dr. Wolfgang Albrecht, Heusenstamm; Kahtan Alkoppa, Kardiologische Praxis Nienburg, Nienburg; Andree Allers, Kardiologie an der Paulikirche, Braunschweig; Tamer Altilar, Kardiologische Gemeinschaftspraxis Gelsenkirchen, Gelsenkirchen; Ayham

Al-Zoebi, Kardiologische Praxis Dr. Al-Zoebi, Wermsdorf; Yuriy Amdur, Praxis Dr. med. Yuriy Amdur, Nürnberg-St. Johannis; Martin Andrassy, Fürst-Stirum-Klinik Bruchsal, Bruchsal; Karl-Friedrich Appel, B. Braun Cardio Zentrum Kassel MVZ GmbH, Kassel;Haydar Aslan, Praxis H. Aslan, Wetzlar; Christoph Axthelm, Hausärztlich-Kardiologisches MVZ „Am Felsenkeller" GmbH, Dresden; Markus Baar, Kardiologische Praxis Northeim, Northeim; Klaus Bachhuber, Praxis Dr. med. Bachhuber, Oberdorf; Fahri Beqa, Herzpraxis Hohenschönhausen, Berlin; Alain Barakat, Gemeinschaftspraxis Alain Barakat und Helene Willems, Duisburg; Marius Barbuia, Ärztezentrum Hammelburg/Dres Edgue/Barbuia/Linke, Hammelburg; Sylvia Baumbach, Gemeinschaftspraxis Dr. med. Sylvia Baumbach und Dr. Elke Reichelt, Apolda; Anselm Bäumer, Praxis für Innere Medizin, Kardiologie, Pneumologie, Köln; Bernd Becker, Praxis am Markt, Essen; Karsten Becker, Praxis Karsten Becker, Wesel; Georg Behnes, Praxis Georg Behnes, Karlsruhe; Roy Ben Chur, Praxis für Allgemeinmedizin, Großes Palais Meiningen – Das Ärzte- und Gesundheitszentrum, Meiningen; Ralf Berg, Praxis im Alten Rathaus, Einzelpraxis Dr. med. R. Berg, Ühlingen-Birkendorf; Klaus Bergmann Klaus, Internistische Schwerpunkt Praxen, Erlangen; Alex-Mihael Berinde, Praxis Dr. Alex Berinde, Neu-Isenburg; Robert Bernat, Westpfalz-Klinikum GmbH - Standort I, Kaiserslautern; Jan Beyer-Westendorf, Universitätsklinikum Carl Gustav Carus TU Dresden, Dresden; Annette Biedermann, Praxis Dr. med. Annette Biedermann, Blankenhain; Konrad Binder Konrad, Praxis Dr. med. Binder, Dreieich-Sprendlingen; Hans-Walter Bindig, Gemeinschaftspraxis Dr.med. Hans-Walter Bindig und Carl-André Bystron, Georgensgmünd; Ralf Birkemeyer, Herzklinik Ulm, Dr. Haerer und Partner, Ulm; Annette Birkenhagen, Praxis Dr. med. Annette Birkenhagen, Stollberg; Erwin Blessing, SRH Klinikum Karlsbad-Langensteinbach GmbH, Karlsbad; Michael Bögel, Praxen Dr. Bögel, Völk und Kollegen, Nürnberg; Mirko Böhme, Praxis Dr. Mirko Böhme, Sulzberg; Andreas Bollmann, Herzzentrum Leipzig GmbH, Leipzig; Dirk

Bonfils, Internistisch-Kardiologische Praxis Kandel, Kandel; Harald Borgmann, Allgemeinmedizinisch Internistische Praxis Dr. med. Harald Borgmann, Radebeul; Ralph Bosch, Cardio Centrum Ludwigsburg Bietigheim, Ludwigsburg; Philipp Boyé, Kardiologische Gemeinschaftspraxis am Park Sanssouci, Potsdam; Bernadett Brado, Praxis für Angiologie und Hämatologie -Dr. med. Bernadett Brado, Heidelberg-Neuenheim; Cornelia Brauer, Praxis Dr. Brauer, Hamburg; Roland Braun, Praxis Dr. med. Roland Braun, Unterschneidheim; Carsten Brinkmann, Praxis für Allgemeinmedizin Dr. med. Carsten Brinkmann, Moormerland-Veenhusen; Karl Bruck, Praxis Dr. med. Karl Bruck, Pirmasens; Markus Buresch, Kardiologische Gemeinschaftspraxis Dr. med. Kösler, Dr. med. Bauer, Dr. med. Buresch und Prof. Dr. med. Niederer, Regensburg; Hermann Burkei, Praxis Dr. med. Hermann Burkei, Kaiserslautern; Carlo Buttron, Praxis Carlo Buttron, Groß Rohrheim; Marco Campo dell' Orto, Sportklinik Bad Nauheim, Bad Nauheim; Matthias Claus, Praxis Dr. Matthias Claus, Berlin-Köpenick; Tillmann Dahme, Universitätsklinikum Ulm, Ulm; Kai-Alexander Dähmlow, Praxis Dr. Kai-Alexander Dähmlow, Murrhardt; Thomas Dengler, SLK-Kliniken Heilbronn GmbH - Klinikum am Plattenwald, Bad Friedrichshall; Michael Denkinger, Agaplesion Bethesda Klinik Ulm, Ulm; Michael Drexler, Cardiopraxis Mainz und Ingelheim GbR - MED Facharztzentrum, Mainz; Rainer Droste, Praxis Dr. med. Rainer Droste, Heilbronn; Mark Dubiel, Praxis Dr. Dubiel, Hamburg; Mustafa Durak, Kardiologische Schwerpunktpraxis, Heidelberg; Michael Dusold, Dres. med. Claudia Wankmüller u. Michael Dusold, Rottweil; Hans-Juergen Dworatzek, Praxis N. Nabo/H.J. Dworatzek, Bad Abbach; Katrin Eberhard, Hausärztlich-Kardiologisches MVZ „Am Felsenkeller" GmbH, Pirna; Hans-Holger Ebert, Gemeinschaftspraxis Dres. med. Stenzel, Ebert & Otto, Riesa; Andreas Ecke, Praxis Dr. med. Andreas Ecke, Rottweil; Egbert Eggers, Kreiskrankenhaus Torgau - Johann Kentmann - gGmbH, Torgau; Dirk Eiser, Gemeinschaftspraxis Dres. med. Pfeffer Sauer Eiser, Lindenberg; Enno Eißfeller, Praxis Dr.

med. Eißfeller, Woellstein; Volker Eissing, MVZ Birkenallee GmbH, Papenburg; Ralf Engelhard, Internistische Gemeinschaftspraxis Dr. med. Engelhard/Dr. med. Wihl, Frankenberg; Fikret Er, Klinikum Gütersloh gGmbH, Gütersloh; Jan Ernstberger, Medizinisches Versorgungszentrum am Küchwald GmbH, Chemnitz; Markus Faghih, Studienzentrum Bocholderstrasse, Dres. med. Faghih/Zühlke, Essen; Peter Falk, Herz-Kreislauf-Klinik Bevensen AG, Bad Bevensen; Dorothea Falkenstein, Praxis Dr. Falkenstein and Dr. Löser, Neustadt; Christian Fechtrup, Praxis für Innere Medizin, Münster; Stephanie Fichtner, Klinikum der Universität München, München; Sven Fischer Sven, Harzklinikum Dorothea Christiane Erxleben GmbH, Quedlinburg; Herbert Fissan, Praxis Dres. Fissan und Werner, Bad Lippspringe; Udo Frädrich, Praxis Dr. med Udo Frädrich, Oberhausen; Peter W. Frank, Praxis Dr. med. Peter W. Frank, Gröbenzell; Dirk Franke Dirk, Institut für Hämostaseologie GmbH, Möser; Jürgen Fritsch, Kardiologie Fritsch - Facharztzentrum am Heilig Geist-Gesundheitszentrum, Köln-Longerich; Emanuel Fritschka, Praxis Prof. Dr. med. Emanuel Fritschka, Bad Brückenau; Andreas Gammel, Praxis Dr. med. Andreas Gammel, Mössingen; Manfred Ganz, Praxis Dr. Rolf Dietzel und Kollegen - ortsübergreifende Gemeinschaftspraxis, Dannenfels; Christoph Garlichs, DIAKO Krankenhaus gGmbH, Flensburg; Oliver Gastmann, Ilm-Kreis-Kliniken Arnstadt-Ilmenau gGmbH, Arnstadt; Johann Christoph Geller, Zentralklinik Bad Berka GmbH, Bad Berka; Sabine Genth-Zotz, Katholisches Klinikum Mainz, Caritas Werk St. Martin Gem. Träger und Betriebsf. GmbH, Mainz; Conrad Genz, Universitätsklinikum Magdeburg A.ö.R., Magdeburg; Uwe Gerbaulet, Praxisgemeinschaft Dr. Biesenbaum und Dr. Gerbaulet, Löhne; Detlef Germann, Hausarztpraxis Anna Lorenz und Detlef Germann, Wetzlar; Tibo Gerriets, Bürgerhospital Friedberg Neurologie, Friedberg; Dirk Glatzel, Praxis Dr. med. Glatzel, Hannover; Andreas Götte, St. Vincenz-Krankenhaus GmbH, Paderborn; Carsten Grabowski, Allgemeinarztpraxis im PueD, Dortmund; Uwe Gremmler, MVZ DaVita Kardiologisches Zentrum Peine GmbH,

Peine; Andreas Greve, Innere Medizin im Gesundheitszentrum, Ahrensburg; C. Michael Gross, Johanniter-Krankenhaus Genthin - Stendal gGmbH, Stendal; Johannes Haas, CIMS UG mbh, Bamberg; Günter Häfele, Praxis für Allgemeinmedizin Dr. med. Günter Häfele, Denzlingen; Dirk Hagemann, Gemeinschaftspraxis Dres. Hagemann, Dürfeld und Breiderhoff, Essen; Andreas Hagenow, Praxis Dr. med. Andreas Hagenow, Elsterwerda; Afif Haj-Yehia, Überörtliche Berufsausübungsgemeinschaft - Dres. med. Beate Zygan, Thomas Reiff, Stephan Linse, Afif Haj-Yehia, Moers; Frank Hamann, Klinikum Konstanz, Konstanz; Bernd Hammer, Gemeinschaftspraxis PD Dr. med. Bernd Hammer und Alexander Schilling, Bechhofen; Detlef Hammerschmidt, Praxis Dr. med. Detlef Hammerschmidt, Bad Krozingen; Tilo Hanfstingl, Gemeinschaftspraxis Hanfstingl, Erlensee; Sabine Hansen, Praxis Dr. med. Sabine Hansen, Unterwellenborn (OT Goßwitz); Stefan Hardt, Kardiologie im Friedrichspalais, Bruchsal; Frank Heckmann, Gefäßzentrum Dr. Heckmann und Kollegen, Neckargemuend; Paul Hegemann, Gemeinschaftspraxis Dr. med. Paul Hegemann/Dr. med. Claus Franck, Ingelheim; Gerd-Ulrich Heinz, Hausärztliche-Internistische Praxis Dr. med. Gerd-Ulrich Heinz, Bergisch-Gladbach/Bensberg; Joachim Heisters, Praxis Dr. med. Joachim Heisters, Kamp-Lintfort Gunter Hergdt, Praxis Dr. med. Gunter Hergdt, Obermichelbach; Petra Herrmann Petra, Praxis Dipl.- Med. Petra Herrmann, Eisenach; Stefan Hetzel, Gemeinschaftspraxis Dres. Hetzel & Grewe-Kemper, Greven; Venesa Hild, Praxis Dr. med. Venesa Hild, Bad Soden-Salmünster; Jörg A. Hintze, Hausarztpraxis und Diabetologische Schwerpunktpraxis Dr. med. Jörg A. Hintze und Dr. med. Martin Grundner, Hainburg; Stefan Hochreuther, Hassberg-Kliniken Haus Hassfurt, Hassfurt; Joachim Hock, Gemeinschaftspraxis Klaus Peter Dausend und Joachim Hock, Oberkirchen; Thomas Hohenstatt, Hausarzt-Praxis - Dr. Thomas Hohenstatt Dr. med. Antonia Dengler, Ursensollen; Andreas Hölscher, Gemeinschaftspraxis im Altstadt-Carree Dres. Simon, Schwuchow, A. u. C. Hölscher, I. Olbert und M. Conze, Fulda; Burkhard Hügl, Marienhaus

Klinikum Neuwied, Neuwied; Hüseyin Ince, Universitätsmedizin Rostock, Rostock; Manja Hubald, Technische Universität Dresden, Dresden; Ulrich Jäck, Praxis Dr. med. Ulrich Jäck, Balingen; Frank-Stephan Jäger, Kardiologie Gemeinschaftspraxis Kassel, Kassel; Sigmund Jakob, Praxis Sigmund Jakob, Weinsberg; Dieter Jänisch-Bernstein, Kardiologische Praxis Waldstraße, Karlsruhe; Gerhard Janßen, Kardiologische Gemeinschaftspraxis am Park Sanssouci, Potsdam; Moritz Jenniches, Praxis Dr. Jenniches, Bad Salzdetfurth; Reinhold Jerwan-Keim, Gemeinschaftspraxis Dr. med. Reinhold Jerwan-Keim, Renate Metz, Dietzenbach; Kurt Jocham, Internistisches Facharztzentrum mit Dialyse Fachbereich Kardiologie - Angiologie, Memmingen; Nils Jonas, Praxis Dr. med. Nils Jonas, Heitersheim; Werner Jung Werner, Schwarzwald-Baar Klinikum Villingen-Schwenningen GmbH, Villingen-Schwenningen; Wolfgang Jungmair, Kardiologische Praxis Dr. med. Wolfgang Jungmair, Bad Homburg; Jan Kähler, Klinikum Herford, Herford; Martin Kajzar, Gemeinschaftspraxis Vogelstang, Mannheim; Tunca Karakas Tunca, Praxis für Innere Medizin Tunca Karakas, Wiesbaden; Dag-Alexander Keilhau, Medizinisches Versorgungszentrum für Innere Medizin, Hamburg; Sebastian Keßler, iKardio, Hürth; Matthias Keim, Kardiologische Gemeinschaftspraxis RT, Reutlingen; Stephan Keldenich, Praxis Dr. Stephan Keldenich, Aachen; Winfried Kessler, Überörtliche Gemeinschaftspraxis - Dres. Kessler, Kessler-Schwigon & Kollegen, Erlangen; Holger Killat, Kardiologische Praxis Holger Killat, Haßloch; Dirk Killermann, DRK-Krankenhaus Grevesmühlen gGmbH, Grevesmühlen; Alexander Klein Alexander, Kardiologische Gemeinschaftspraxis Dres Klein & Neumann, Stuttgart; Karin Klein Karin, Gemeinschaftspraxis Dres. Klein, Schreiber & von Petzinger-Kruthoff, Remagen; Norbert Klein, Klinikum St. Georg gGmbH, Leipzig; Waldemar Klimek, Dr. Klimek MVZ GmbH, Neuburg an der Donau; Carsten Klingner, Universitätsklinikum Jena, Jena; Martin Klutmann, Praxis für Kardiologie Aachen GbR, Aachen; Steffen Kolschmann, Kardiologen am Fetscherplatz - Gemeinschaftspraxis für

Kardiologie Dr. med. Michael Günther & Dr. med. Steffen Kolschmann, Dresden; Andreas Kopf, Praxis Dr. med. Andreas Kopf, Berlin; Vivion Koppatsch, Praxis Dr. med. Vivion Koppatsch, Bad Buchau; Grigorios Korosoglou, GRN-Klinik Weinheim, Weinheim; Jürgen Krafft, Praxis Jürgen Krafft, Zirndorf; Fabian Krämer, Zentrum für Prävention und Rehabilitation, Praxisgemeinschaft Dr. med. Ulrich Overhoff, Dr. med. Fabian Krämer, Siegen; Roland Krämer, Hausärztliche Praxisgemeinschaft Dr. med. Roland Krämer und Dr. med. Peter Rauh, Berlin-Neukölln; Daniel Kretzschmar, Universitätsklinikum Jena, Jena; Stephan Kreutz, Praxis Dr. med. Stephan Kreutz, Maxdorf; Manuel Kronschnabl, Gemeinschaftspraxis Dr. med. Bruno Kronschnabl, Dr. med. Manuel Kronschnabl, Sonja Römer-Kronschnabl, Regen; Thomas Kuhl, Praxis für Kardiologie, Gesundheitsförderung und Prävention, Dormagen; Christian Kühne, Kardiologische Gemeinschaftspraxis Dres. Loebe, Weißbrodt, Leipzig; Jörg Kynast, Kardiologie Alsterquelle, Praxis Dr. Jörg Kynast, Henstedt-Ulzburg; Matthias Läger, Praxis Dr. Matthias Läger, Merseburg; Felix Lampe, Kreiskrankenhaus Emmendingen, Emmendingen; Jörg Langel, Praxis für Kardiologie Dr. med. Jörg Langel, Gera; Wasilios Lazaridis, Innere am Stadtpark Dr. med. Martin Laser, Nürnberg; Hassan Lebbed, Herzcarre Kardiologie Bad Homburg, Bad Homburg; Karim Sven Liem, Kardiologie am Rotkreuzplatz, München; Sven Linzbach, Kardiocentrum Frankfurt an der Klinik Rotes Kreuz, Frankfurt; Josef Lißmann, Praxis Dr. med. Josef Lißmann, Homburg; Hugo Loeber, Internistische Praxis Hugo Loeber, Weiskirchen; Thomas Loeseken, Praxis für Allgemeinmedizin Dr. Loeseken, Rohrberg; Christian Loges, Kariologische Gemeinschaftspraxis Mosbach Dres. Haney/Gehrig/Loges, Mosbach; Jan Lokies, Gemeinschaftspraxis Dres. Frank Eickhoff und Jan Lokies, Berlin; Sabine Lorenz, Praxis Dr. med. Sabine Lorenz, Bochum; Stephan Lüders, St. Josefs-Hospital, Cloppenburg; Norbert Ludwig, Gemeinschaftspraxis Prof. Dr. med. Ludwig und Dr. med. Honl, Willich-Anrath; Thomas Ludwig, Praxis Dr. med. Thomas Ludwig, Farchant; Carola Lüke,

Praxisgemeinschaft Jerichow, Schönhausen; Erik May, MVZ im Rolshover Hof AmKaRe Poll - Dres. Gysan, Heinzler, May GbR, Köln; Sony Mayerheim, MVZ Gropiusstadt, Berlin; Eckhard Meisel, Praxisklinik Herzkreislauf am Wasa-Platz, Dresden; Frank Menzel, Praxis für Kardiologie und Angiologie Dr. med. Frank Menzel, Dessau-Roßlau; Charlotte Metreveli-Wiese, Praxis Drs Wiese/Metreveli-Wiese, Osnabrück; Michael Metze, Universitätsklinikum Leipzig AöR, Leipzig; Holger Michel, Gemeinschaftspraxis Michel und Leffler, Lutherstadt Eisleben; Veselin Mitrovic, Kerckhoff-Klinik Forschungsgesellschaft mbH, Bad Nauheim; Christian Mahnkopf, Klinikum Coburg GmbH, Coburg; Heiner Müller, Berufsausübungsgemeinschaft Dres. med. Kolitsch/Müller, Katzhütte; Karsten Müller, Praxis Dr. med. Karsten Müller, Gräfenhainichen; Stefan Müller, Kardiologische BAG - Dr. med. Glatthor, Dr. med. Müller, Dr. med. Schlotterbeck und Dr. med. Trompler, Weingarten; Walter Müller, Praxis für Herz-Kreislauf Erkrankungen, Flensburg; Mathis Münchbach, Praxis Prof. Staedt/Dr.med. M. Münchbach, Speyer; Mohammed Natour, Heidelberger Praxisklinik für Innere Medizin, Kardiologie und Pneumologie, Heidelberg; Jens Nettelrodt, Herz-/Gefäßpraxis Morsbach am Teufelsbrunnen, Morsbach; Peter Nordbeck, Universitätsklinikum Würzburg, Würzburg; Christian Oehlwein, Praxis Dr. med. Christian Oehlwein, Gera; Eva Olesch, Überörtliche Berufsausübungsgemeinschaft - Dres. med. Knöbel/Pelz-Knöbel/Olesch/von Hassel/Spaltmann, Straubing; Christian Opitz, DRK Kliniken Berlin Westend, Berlin; Karl Josef Osterziel, Prof. Dr. med. Karl Josef Osterziel und Peter Wittmann Oberpfalz GbR, Amberg; Ulrich Overhoff, Zentrum für Prävention und Rehabilitation, Praxisgemeinschaft Dr. med. Ulrich Overhoff, Dr. med. Fabian Krämer, Siegen; Thomas Pätzold, Praxis Dr. med. Thomas Pätzold, Rudolstadt; Andrea Perne, INNNERE MED3 – Praxis für Innere Medizin, Walldorf; Anton Peter, Gemeinschaftspraxis Dres. Franz Gaim & Peter Anton, Aiterhofen; Hannelore Pitule, Praxis Dr. med. Hannelore Pitule, Ludwigshafen; Alexander Plehn, Praxisklinik Salzatal, Salzmünde; Karsten Pohle,

Krankenhaus Martha-Maria Nürnberg, Nürnberg; Martin Prohaska, Gemeinschaftpraxis Dr. med. Martin Prohaska und Dr. med. Felix Schulte, Mühldorf; Roland Prondzinsky, Carl-von-Basedow-Klinik Saalekreis GmbH, Merseburg; Ursula Rauch-Kröhnert, Charité - Universitätsmedizin Berlin - Campus Benjamin Franklin, Berlin; Matthias Regenfus, Kardiologie Dr. med. Matthias Regenfus, Nürnberg; Günter Rehling, Gemeinschaftspraxis Dres. Michael Eis und Günter Rehling, Sand am Main; Gernot Reimann, Klinikum Dortmund gGmbH, Dortmund; Claus-Michael Reimers, Hausarztpraxis-Offenbach, Offenbach; Helmut Renz, Gemeinschaftspraxis Vogelstang, Mannheim;; Jean Rieber, Kardiologische Praxis Dres. Rieber and Ganschow, Leinfelden-Echterdingen; Werner A. Rieker, Praxisgemeinschaft Rankestrasse, Berlin; Oliver Ritter, Städtisches Klinikum Brandenburg, Brandenburg an der Havel; Sabine Roeder, Kardiologische Praxis Dr. med. Sabine Roeder, Berlin; Michael Rother, Ärztehaus Kastanienallee - Praxis Dr. Michael Rother, Strausberg; Stefan Sack, München Klinik gGmbH - Klinikum Neuperlach, München; Thomas Sanner, Internistische Gemeinschaftspraxis Thomas A. Sanner & Uwe Wojts, Weiterstadt; Alexander Sattler, Internistische Gemeinschaftspraxis Biedenkopf, Biedenkopf; Regina Schapfeld, Praxis Dres. Rolfes & Schapfeld, Egelsbach; Karl-Heinz Schermaul, Praxis für Innere Medizin und Kardiologie - Dipl.-Med. Karl-Heinz Schermaul, Chemnitz; Olive Scheuermann, Gemeinschaftspraxis Dres. Persicke und Scheuermann, Kornwestheim; Axel Schlitt, Paracelsus-Harz-Klinik Bad Suderode, Quedlinburg; Michael Schmid, Kardio-IN - Kardiologische Gemeinschaftspraxis Ingolstadt, Ingolstadt; Andor Schmidt, Praxis Dr. med. Lutz Kröning, Offenbach; Fabian Schmidtler, Praxis Dr. med. Fabian Schmidtler, Burghausen; Astrid Schmidt-Reinwald, Praxis Dr. med. Astrid Schmidt-Reinwald, Trier; Ulrich Schmidt-Rosenbaum, Allgemeinarztpraxis im PueD, Dortmund; Detlef Schmitz, Praxis Dr. Schmitz, Erlenbach a. Main; Karl-Heinz Schnaibel, Praxis Dr. med. Karl-Heinz Schnaibel, Baden-Baden; Reinhold Schneider, Praxis Dr. med. Reinhold Schneider und

Christine Schneider, Wetzlar-Naunheim; Stefan Schneider, Praxis Dr. med. Stefan Schneider, Rieschweiler-Mühlbach; Norbert Schöll, MCN Medic-Center Nürnberg GmbH, Nürnberg; Susanne Schöller, Praxis Dr. med. Susanne Schöller, Horb am Neckar; Hans-Jürgen Scholz, Praxis Dr. H.-J. Scholz, Schwetzingen; Norbert Schön, Kardiologie Mühldorf am Inn Dr. med. Norbert Schön, Mühldorf am Inn; Andreas Schreckenberg, Internistische Praxisgemeinschaft, Weyhe; Jörg Schulze, Praxisgemeinschaft Jerichow, Jerichow; Matthias Schulze Matthias, Asklepios Klinikum Schwalmstadt, Schwalmstadt; Henrik Schumann, Gemeinschaftspraxis Dres. med. Hartwig & Henrik Schumann, Nidderau; Bernd Schüttrumpf, Gemeinschaftspraxis Amin Satli und Dr. med. Bernd Schüttrumpf, Sarstedt; Bernd Schütz, Gemeinschaftspraxis Erdmann Schütz, Luenen; Ralf-Joachim Schwab, Gemeinschaftspraxis Dres. med. Barth, Birkofer, Müller-Hörner, Schwab, Nürnberg; Kurt Schwabe, Praxis am Landratspark, Bad Segeberg; Harald Schwacke, Diakonissen-Stiftungs-Krankenhaus Speyer, Speyer; Toralf Schwarz, INFO-MED Leipzig GmbH, Zwenkau; Thomas Segiet, Diabetologische Schwerpunktpraxis Dres. Segiet, Gleixner und Bode, Speyer; Jaroslaw Sek, Kardiologische Gemeinschaftspraxis Dres. Kaltofen/Schubert/Gerner/Jurowsky, Chemnitz; Jörg Simon, Gemeinschaftspraxis Dres. Simon, Schwuchow, A. u. C. Hölscher, I. Olbert und M. Conze, Fulda; Gregor Simonis, Zentrum für klinische Prüfungen in der Facharztzentrum Dresden-Neustadt GbR, Dresden; Ilka Simon-Wagner, Praxis am Oberen Tor Dr. med. Ilka Simon-Wagner, Lichtenfels; Jaswant Singh, St. Elisabeth Krankenhaus Jülich, Jülich; Hassan Soda, Rhön-Klinikum AG, Rhön-Klinikum Campus Bad Neustadt, Bad Neustadt a. D. Saale; Merima Spahic, Klinikum Stuttgart, Stuttgart; Ulrike Spengler, Praxis Dr. med. Ulrike Spengler, Borsdorf-Panitzsch; Johannes Spormann, Praxisgemeinschaft Oldesloer Strasse, Dr. med. Johannes Spormann, Hamburg; Alexander Stadelmann, Kardiologie am Weißen Turm Dr. med. Alexander Stadelmann, Nürnberg; Raphael Steger, Praxis Raphael Steger, Deggenhausertal; Kerstin

Steinbach, Dipl.- Med. Kerstin Steinbach, Freital; Stefan Steiner, Herz- und Kreislauf-Zentrum Rotenburg a. d. Fulda, Rotenburg a. d. Fulda; Heiko Stellmach, Kardiologische Praxis Dr. med. Heiko Stellmach, Chemnitz; Matthias Stratmann, Kardiologische PraxisGemeinschaft Kampstraße, Dortmund; Tim Süselbeck, Kardiologische Praxisklinik Ludwigshafen, Ludwigshafen; Jens Taggeselle, Kardiologische Praxis Dr. Jens Taggeselle, Markkleeberg; Alexander Tecl, Praxis Dr. Robert Tecl & Kollegen, Schriesheim; Magdalena ten Hoevel, Gemeinschaftspraxis Ewa Drzewinska und Dr. Magdalena ten Hoevel, Hamminkeln; Andre Terhorst, Praxis Andre Terhorst, Hamminkeln; Dierk Thomas, Universitätsklinikum Heidelberg, Heidelberg; Svetlana Tlechas-Tkatsch, Praxis S. Tlechas-Tkatsch, Potsdam; Fabian Tölle, Praxis Dres. Fabian Tölle und Bernadette Jauch GbR, Flieden; Ramesh Tripathi, Praxis Dr. Tripathi, Wuppertal; Steffen Tröger, Kardiologische Praxis Dr. med. Steffen Tröger, Chemnitz; Christian Ukena, Universitätsklinikum des Saarlandes, Homburg; Achim Ulmer, Praxis Dr. med. Achim Ulmer, Ludwigsburg; Thomas Vogtmann, Kardiologische Gemeinschaftspraxis am Park Sanssouci, Potsdam; Jan-Gerrit Voigt, Praxis Dr. med. Jan-Gerrit Voigt, Dorsten; Wolfgang C. G. von Meißner, Gemeinschaftspraxis Dres. Seitz und von Meißner, Baiersbronn; Reinhold Vormann, Praxisgemeinschaft Dr. H. Boeneke/P. B. Vormann, Lienen - Kattenvenne; Wolfram Wagner, Hausarztpraxis W. Wagner und Dr. E. Hofmann, Stegaurach; Ascan Warnholtz, Kardiologische Praxis Dr. Tauchert und Prof. Dr. Warnholtz, Griesheim; Frank Warzok, Kardiologische Praxis Dr. med. Frank Warzok, Gotha; Jörn Weckmüller, Praxis Dr. med. Jörn Weckmüller, Lübeck; Joachim Weil, SANA Klinik Lübeck, Lübeck; Michael Weisbach, Kardiologie am Tibarg, Hamburg; Georg Weiß, Praxis Georg Weiß, Weingarten/Pfalz; Philipp Wende, Praxis Dr. Wende, Aschaffenburg; Thomas Wetzel, Gemeinschaftspraxis für Kardiologie Dr. med. Walter Willgeroth und Dr. med. Thomas Wetzel, Dortmund; Georg Weyers, KardioPraxis - Gemeinschaftspraxis für Herz-Kreislauferkrankungen, Bergisch

Gladbach; Cosmas Wildenauer, Praxis Dr. med. Wildenauer, Bad Brückenau; Andreas Wilke, Kardiologische Praxis Papenburg Dr. Andreas Wilke & Dr. Andrej Malazhavy, Papenburg; Ulrich Windstetter, Kardiologische Gemeinschaftspraxis an der Universität Dr. med. Wauer und Dr. med. Windstetter, München; Anne Winkelmann Anne, Kardiologische Facharztpraxis Dr. Anne Winkelmann, Berlin; Jan Winkler, Kardiologische Praxis Winkler, Hohenstein-Ernstthal; Christina Winkler Christina, MVZ MP Saaletal, Saalfeld; Bernhard Witzenbichler, HELIOS Amper-Klinikum Dachau, Dachau; Franz Wolf, Praxis Dres. Wolf, Straubing; Ulrich Wolf, Parkkardiologie, Stahnsdorf; Wojciech Zawalski, Gemeinschaftspraxis Dr. Unger und Zawalski, Giengen an der Brenz; Hans-Hermann Zimny, Praxis Dr. med. Zimny, Bad Pyrmont; Rainer J. Zotz, Marienhaus Klinikum Eifel Bitburg, Bitburg; Christian Zugck, Internistische Gemeinschaftspraxis Steiner Thor, Straubing; Uwe Zwettler, Atos Klinik Heidelberg, Heidelberg.

Ireland

Mazen Al Alawi, Our Lady's Hospital, Navan; Godfrey Aleong, Letterkenny General Hospital, Letterkenny; David Burke, Beacon Hospital, Dublin; Michael Conway, St. Luke's General Hospital, Kilkenny; Eamon Dolan, Connolly Hospital Blanchardstown, Dublin; Liam Glynn, Ballyvaughan Medical Centre, Ballyvaughan; Mark Laher, Blackrock Clinic, Dublin; Peter Kearney, Cork University Hospital, Cork; Peter Kelly, Mater Misericordiae University Hospital, Dublin; Thomas Kiernan, University Hospital Limerick, Co Limerick; Charles McCreery, St Vincent's University Hospital, Dublin; Ken McDonald, St Vincent's University Hospital, Dublin; Ross Murphy, St James's Hospital, Dublin; Darren Mylotte, University Hospital Galway, Galway; Patrick Owens, Waterford Regional Hospital, Waterford; Richard Sheahan, Beaumont Hospital, Dublin.

Italy

Raffaele De Caterina, Azienda Ospedaliero Universitaria Pisana Cisanello, Pisa; Serafina Valente, Ospedale Le Scotte di Siena, Siena; Massimo Piepoli, Ospedale "Guglielmo da Saliceto", Piacenza; Enrica Petruccelli, Ospedale San Giacomo di Monopoli, Monopoli; Angela Beltrame, San Donà del Piave Hospital, ULSS4, Veneto Orientale; Giulia Renda, ASL n. 2 Lanciano Vasto Chieti Ospedale SS. Annunziata di Chieti 3110 Treviso, Chieti; Nicola De Luca, Ospedale Civile Spirito Santo, Pescara; Leonardo Paloscia, Ospedale Civile Spirito Santo, Pescara; Claudio Ferri, Ospedale San Salvatore, L'Aquila; Cosimo Napoletano, Ospedale Civile Giuseppe Mazzini, Teramo; Marco Fabio Costantino, AOR San Carlo, Potenza; Michele Antonio Clemente, ASM Matera Presidio OspedalieroMadonna Delle Grazie, Matera; Luigi Anastasio, Azienda Sanitaria Provinciale di Vibo Valentia, Presidio Ospedaliero G. Jazzolino, Vibo Valentia; Ciro Indolfi, Azienda Ospedaliera Universitaria Mater Domini, Catanzaro; Angela Sciacqua, A.O.U. Mater Domini, Catanzaro; Giuseppe Bencardino, Casa di Cura Tricarico Rosano, Belvedere Marittimo; Francesco Solimene, Casa di Cura Privata Montevergine S.p.A., Mercogliano; Emilio di Lorenzo, Azienda Ospedaliera di Rilievo Nazionale e di Alta Specialità San Giuseppe Moscati, Avellino; Marino Scherillo, Azienda Ospedaliera Gaetano Rummo, Benevento; Alfredo Vetrano, Azienda Ospedaliera di Caserta, Sant'Anna e San Sebastiano, Caserta; Antonio D'Onofrio, Ospedale Monaldi - AOS dei Colli, Napoli; Gerardo Nigro, Ospedale Monaldi - AOS dei Colli, Napoli; Gerolamo Sibilio, Ospedale Santa Maria delle Grazie, Pozzuoli; Raffaele Sangiuolo, Ospedale Buon Consiglio Fatebenefratelli, Napoli; Bernardino Tuccillo, ASL NA 1 Presidio Ospedaliero Santa Maria di Loreto Mare, Napoli; Giuseppe Stabile, Clinica Mediterranea SPA, Napoli; Paolo Tammaro, Ospedale S. Giovanni Bosco ASL Città di Torino, Torino; Pasquale Guarini, Casa di cura privata Villa dei Fiori srl, Acerra; Giuseppe Bruzzese, ASL NA 1 Centro P.O. dei Pellegrini, Napoli; Ciro Mauro, A.O. di Rilievo Nazionale A. Cardarelli e A.O.R.N.

Santobono-Pausilipon, Napoli; Michele Roberto Di Muro, A.O.U. OO.RR. San Giovanni di Dio Ruggi d'Aragona, Salerno; Antonio Rapacciuolo, Azienda Ospedaliero Universitaria Federico II, Napoli; Marcello Bertorelli, Ospedale Santa Maria, Borgo di Val di Taro; Letizia Riva, Ospedale Maggiore di Bologna AUSL di Bologna, Bologna; Stefano Urbinati, Ospedale di Bellaria, Bologna; Eugenio Cosentino, Azienda Ospedaliero Universitaria di Bologna Policlinico Sant'Orsola - Malpighi, Bologna; Igor Diemberger, Azienda Ospedaliero Universitaria di Bologna Policlinico Sant'Orsola - Malpighi, Bologna; Stefano Baccarini, P.O. Vaio Fidenza - AUSL Parma, Fidenza; Roberto Carletti, Ospedale Morgagni - Pierantoni, Forlì; Giancarlo Piovaccari, Ospedale "Infermi" di Rimini - AUSL Romagna, Rimini; Alessandro Corzani, Ospedale M. Bufalini, Cesena; Alessandro Fucili, Arcispedale S. Anna, Ferrara; Giuseppe Boriani, Policlinico di Modena, Modena; Mauro Zennaro, Nuovo ospedale Civile Sant Agostino-Estense, Modena; Claudio Fresco, Azienda Ospedaliero Universitaria, Santa Maria della Misericordia, Udine; Luisa Mattei, Ospedale di Gorizia, Gorizia; Laura Perale, Presidio Ospedaliero di Latisana e Palmanova, Latisana; Carmine Mazzone, Ospedale Maggiore Centro Cardiovascolare, Trieste; Elisabetta Ricottini, Policlinico dell'Università Campus Bio-Medico di Roma, Roma; Giuseppe Patti, Policlinico dell'Università Campus Bio-Medico di Roma, Roma; Guido Melillo, Istituto Dermopatico dell'Immacolata IDI-IRCCS, Roma; Massimo Volpe, A.O. Sant'Andrea, Roma; Francesco Fedele, Policlinico Umberto I, Roma; Raffaele Quaglione, Policlinico Umberto I, Roma; Pasquale Pignatelli, Policlinico Umberto I, Roma; Matteo Ruzzolini, Ospedale "San Giovanni Calibita" Fatebenefratelli Isola Tibertina, Roma; Furio Colivicchi, Ospedale San Filippo Neri- ASL Roma E, Roma; Gian Francesco Mureddu, Ospedale San Giovanni Addolorata, Roma; Filippo Maria Sarullo, Ospedale Buccheri La Ferla Fatebenefratelli, Palermo; Leonardo Calò, Ospedale Policlinico Casilino-ASL Roma B, Roma; Fabrizio Ammirati, Ospedale G.B. Grassi-ASL Roma D, Roma; Letizia Maria Cupini, Ospedale

S.Eugenio-ASL Roma C, Roma; Natale Di Belardino, Ospedale di Anzio- Nettuno-ASL Roma H, Anzio -RM; Maurizio Menichelli, Ospedale Fabrizio Spaziani-ASL di Frosinone, Frosinone; Francesco Caprioglio, Ospedale San Bortolo di Vicenza, Vicenza; Francesca Alfonsi, Ospedale SS. Trinità-Sora, Sora; Roberto Pola, Fondazione Policlinico Universitaria "A.Gemelli", Roma; Stefano Pardi, Ospedale Santa Scolastica di Cassino-ASL di Frosinone, Cassino; Massimo Zoni Berisso, Ospedale Padre Antero Micone di Sestri Ponente - ASL 3 Genovese, Genova; Pietro Ameri, IRCCS. A.O.U. San Martino IST, Genova; Mattia Laffi, Ospedale Villa Scassi ASL GE 3 Genovese, Genova; Livia Paonessa, Ospedale S. Corona ASL 2 Savonese, Pietra Ligure; Rossella Petacchi, ASL5 Liguria Ospedale San Bartolomeo, Sarzana; Attilio Iacovoni, Ospedale Papa Giovanni XXIII - ASST Papa Giovanni XXIII, Bergamo; Nicoletta De Cesare, Policlinico San Marco, Zingonia; Andrea Cafro, ASST Bergamo EST Ospedale Bolognini, Seriate; Andrea Capoferri, ASST Bergamo OVEST Ospedale "Treviglio Caravaggio", Treviglio; Savina Nodari, Ospedale Spedali Civili- ASST Spedali Civili, Brescia; Claudio Cuccia, Fondazione Poliambulanza Istituto Ospedaliero, Brescia; Ilaria Romano, Ospedale Bassini - ASST Milano Nord, Cinisello Balsamo; Giuseppe Cattafi, Grande Ospedale Metropolitano di Niguarda, Milano; Giuseppe De Angelis, Ospedale di Rho - ASST Rhodense, RHO; Carlo Piemontese, Ospedale S. Anna - ASST Lariana, San Fermo della Battaglia; Roberta Gestra, ASST Valtellina e Alto Lario- Ospedale di Sondrio, Sondrio; Maurizio Eugenio Landolina, Ospedale Maggiore di Crema - ASST di Crema, Crema; Enrico Passamonti, Ospedale di Cremona - ASST di Cremona, Cremona; Albino Reggiani, Ospedale di Mantova - ASST di Mantova, Mantova; Daniele Nicolis, Ospedale Pieve di Coriano -ASST di Mantova, Pieve Di Coriano; Giuseppe Di Tano, Ospedale di Oglio Po-ASST di Cremona, Vicomoscano; Piergiuseppe Agostoni, Centro Cardiologico Monzino, Milano; Claudio Tondo, Centro Cardiologico Monzino, Milano; Manuela Cireddu, Ospedale San Raffaele, Milano; Alberto Margonato, Ospedale San

Raffaele, Milano; Paolo Bucciarelli, Fondazione IRCCS Ca’ Granda Ospedale Maggiore Policlinico, Milano; Corrado Lodigiani, Istituto Clinico Humanitas, Milano; Elena Piazzi, ASST Monza- Ospedale San Gerardo, Monza; Giuseppe Gallone, Policlinico di Monza, Monza; Luigi Oltrona Visconti, Fondazione IRCCS Policlinico San Matteo, Pavia; Sabino Iliceto, Azienda Ospedaliera di Padova, Padova; Francesco Castagna, Ospedale Classificato "Sacro Cuore - Don Calabria" di Negrar, Negrar; Valeria De Micheli, ASST Lecco - P.O. di Merate, Lecce; Daniele Nassiacos, Ospedale di Saronno - ASST Valle Olona, Saronno; Marina Diomedi, U.O.C. Stroke Unit Policlinico Tor Vergata, Roma; Gianfranco Parati, Istituto Scientifico Ospedale San Luca, Milano; Federico Guerra, A.O.U. Ospedali Riuniti Umberto I - G.M. Lancisi – G. Salesi, Ancona; Gian Piero Perna, A.O.U. Ospedali Riuniti Umberto I - G.M. Lancisi – G. Salesi, Ancona; Roberto Antonicelli, Istituto Nazionale di Riposo e Cura per Anziani IRCCS, Ancona; Antonio Mariani, Ospedale di Senigallia, Senigallia; Pietro Scipione, Ospedale Fabriano -ASUR Marche, Fabriano; Cristina D'Ambrosio, Ospedale F. Veneziale, Isernia; Patrizia Noussan, Ospedale San Giovanni Bosco, Torino; Gaetano Senatore, Ospedale Civile di Ciriè, Ciriè; Riccardo Riccardi, Ospedale Edoardo Agnelli, Pinerolo; Ferdinando Varbella, Ospedale Degli Infermi di Rivoli, Torino; Sergio Agosti, Ospedale San Giacomo, Novi Ligure; Roberto Santi, A.O. Nazionale SS. Antonio Biagio e Cesare Arrigo, Centro Emostasi e Trombosi, Alessandria; Carla Giustetto, A.O.U. Città della Salute e della Scienza di Torino, Presidio ospedaliero Molinette, Torino; Virginia Bolzani, Ospedale Maggiore della Carità, Novara; Alessandro Lupi, Ospedale Castelli di Verbania, Verbania; Vito Sollazzo, Ospedale Civile Ospedale Masselli-Mascia, San Severo; Natale Daniele Brunetti, Azienda Ospedaliero Universitaria “Ospedali Riuniti” di Foggia, Foggia; Graziano Riccioni, Ospedale San Camillo De Lellis, Manfredonia; Michele Balsamo, Ospedale San Camillo De Lellis, Manfredonia; Michele Cannone, Ospedale Civile Bonomo, Andria; Giuseppe Modugno, Ospedale Civile Vittorio

Emanuele II, Bisceglie; Mariano Rillo, Casa di cura Villa Verde, Taranto; Armando Liso, Casa di cura Città di Lecce, Lecce; Mariella Callerame, Ospedale Vito Fazzi, Lecce; Francesca Barba, Azienda Ospedaliera "Cardinale G. Panico", Tricase; Massimo Trianni, Cittadella Della Salute (ex Fazzi), Lecce; Giuseppina De Benedittis, Cittadella Della Salute (ex Fazzi), Lecce; Antonio Francesco Amico, San Giuseppe Copertino, Galatina; Pasquale Caldarola, Ospedale San Paolo, Bari; Paolo Colonna, Azienda Ospedaliera Universitaria Consorziale Policlinico Bari, Bari; Stefano Favale, Azienda Ospedaliera Universitaria Consorziale Policlinico Bari, Bari; Massimo Grimaldi, Ospedale Generale Regionale "F. Miulli", Acquaviva delle Fonti; Andrea Passantino, Istituto Scientifico Ospedale Maugeri di Cassano delle Murge, Cassano delle Murge; Marco Corda, Ospedale Brotzu di Cagliari, Cagliari; Luigi Meloni, AOU Cagliari-Presidio San Giovanni di Dio, Cagliari; Caterina Gaddeo, Ospedale San Francesco di Nuoro, Nuoro; Pierfranco Terrosu, Ospedale SS. Annunziata, Sassari; Corrado Tamburino, Ospedale Ferrarotto, Catania; Michele Gulizia, Garibaldi-Nesima, Catania; Salvatore Guarnera, Centro Cuore Morgagni, Pedara; Marco Contarini, Ospedale Umberto I, Siracusa; Giovanni Licciardello, Ospedale E. Muscatello, Augusta; Carmelo Fossi, Ospedale "Gravina e Santo Pietro " Caltagirone, Caltagirone; Nicola Adragna, Villa Maria Eleonora, Palermo; Giuseppe Caramanno, S. Giovanni di Dio, Agrigento; Gaspare Rubino, Ospedale Paolo Borsellino, Marsala; Vincenzo Cirrincione, Ospedali Riuniti Villa Sofia-Cervello, Presidio Ospedaliero C.T.O., Palermo; Giuseppe Andò, A.O. Universitaria Policlinico, G. Martino Messina, Messina; Rossella Marcucci, A.O.U. Careggi, Firenze; Niccolò Marchionni, A.O.U. Careggi, Firenze; Nazario Carrabba, A.O.U. Careggi, Firenze; Vieri Vannucchi, Ospedale Santa Maria Nuova di Firenze, Firenze; Massimo Milli, Ospedale Santa Maria Nuova di Firenze, Firenze; Giuseppe Arena, Ospedale Civile USL1 di Massa Carrara, Massa Carrara; Umberto Baldini, Ospedale di Livorno, Livorno; Maria Grazia Bongiorni, Nuovo Ospedale Santa Chiara, Pisa; Alessandro Costoli,

P.O. di Grosseto, Grosseto; Leonardo Bolognese, Ospedale San Donato di Arezzo, Arezzo; Roberto Cemin, Ospedale di Bolzano, Bolzano; Giancarlo Agnelli, Santa Maria della Misericordia, Perugia; Claudio Cavallini, Santa Maria della Misericordia, Perugia; Claudia Bartolini, Media Valle del Tevere, Perugia; Roberto Verlato, Ospedale di Camposampiero - Ulss 6 Euganea, Camposampiero; Leopoldo Pagliani, Ospedale Riabilitativo di Motta di Livenza, Motta di livenza; Maurizio Anselmi, ULSS 9 Scaligera Presidio Ospedaliero Fracastoro, San Bonifacio; Francesco Di Pede, Ospedale San Donà U.O. Cardiologia, San Donà Di Piave; Ornella Barbato, Ospedale Civile di Mirano, Mirano; Salvatore Saccà, Ospedale Civile di Mirano, Mirano; Antonino Mazzone, Ospedale Civile di Legnano, U.O. Medicina Interna, Legnano; Marcello Piacenti, Fondazione Toscana G. Monasterio, Dip. Elettrofisiologia Interventistica, Pisa; Filippo Risaliti, Nuovo Ospedale di Prato - Santo Stefano, Dip. Area Medica, Prato; Francesco Amico, A.O. Cannizzaro - U.O. Cardiologia, Catania; Eduardo Rebulla, Casa di Cura Candela - Dip. Cardiologia, Palermo; Daniela Aschieri, Ospedale Unico della Valtidone - U.O. Cardiologia - Dip. Emergenza Urgenza, Castel San Giovanni; Antonio Sanmartino, Ospedale Humanitas Gardenigo - U.O. Cardiologia, Torino; Luigi Caliendo, P.O. Santa Maria della Pietà - U.O. Cardiologia-UTIC, Nola; Luigi Di Lorenzo, Ospedale S. Rocco, Sessa Aurunca; Antonio Cittadini, Direttore C.R.I.B. - Università degli Studi Federico II di Napoli, Napoli; Roberto Valle, Ospedale di Chioggia, Chioggia; Fausto Rigo, Ospedale dell'Angelo, Mestre; Mauro Feola, Ospedale di Fossano - U.O. Riabilitazione Cardiologica, Fossano; Cesare Storti, Istituto Città di Pavia - Cardiologia, Pavia; Giovanni Sarli, Osp. Frascati - Cardiologia, Frascati; Simona D'Orazio, Ospedale Santa Margherita – Valdichiana, Cortona; Roberto Pontremoli, IRCCS. A.O.U. San Martino IST, Genova; Maurizio Ziliotti, P.O. Vaio Fidenza - AUSL Parma, Fidenza.

The Netherlands

Harry Crijns, Maastricht University Medical Center, Maastricht; Wilhelm ten Holt, Ziekenhuis Amstelland, Amstelveen; Saman Rasoul, Zuyderland Medisch Centrum, Heerlen; Jacob van Eck, Jeroen Bosch Ziekenhuis, Carolus, Den Bosch; Mathijs Pieterse, Stichting Cardiologie Amsterdam, Amsterdam; Jonas De Jong, Onze Lieve Vrouwe Gasthuis, Locatie Oost, Amsterdam; R. Vromans, Maasziekenhuis Pantein, Boxmeer; J.F. Vanderheijden, UMC Utrecht, Utrecht; Frank den Hartog, Gelderse Vallei Ziekenhuis, Ede; Richard Folkeringa, Medisch Centrum Leeuwarden, Leeuwarden; Joris de Groot, Amsterdam UMC, Amsterdam; Salah Said, Ziekenhuisgroep Twente, Hengelo; Ayten Yilmaz, Maasstad Ziekenhuis, Rotterdam; Coenraad Van der Zwaan, Ziekenhuis Rivierenland, Tiel; Remco Nijmeijer, Ziekenhuis Tjongerschans, Heerenveen; C.P. Allaart, Amsterdam UMC, Locatie VUMC, Amsterdam; Jur ten Berg, St. Antonius Ziekenhuis, Nieuwegein; Martin Hemels, Rijnstate, Arnhem; M. A Brouwer, Radboud Nijmegen, Nijmegen; Machiel van de Wetering, BovenIJ Ziekenhuis, Amsterdam; Francisco Prins, Elkerliek Ziekenhuis, Helmond; Lucas Van Beek, Ziekenhuis De Sionsberg, Dokkum; R.F Veldkamp, HMC Westeinde, Den Haag; Ruud van de Wal, Bernhoven Uden, Uden; Ferdinant van Nooijen, Dijklander Ziekenhuis, Locatie Purmerend, Purmerend; Erno van Nes, Laurentius Ziekenhuis, Roermond; Tjeerd Romer, Alrijne Ziekenhuis, Leiden; Joan Meeder, VieCuri Medisch Centrum, Venlo; Eric Viergever, Groene Hart Ziekenhuis, Gouda; Iris Westendorp, Rode Kruis Ziekenhuis, Beverwijk; T.G.M. van Delft, Havenziekenhuis, Rotterdam; Marcus van der Linden, Franciscus Vlietland, Schiedam; DPW Beelen, IJsselland Ziekenhuis, Capelle aan den IJssel; Lex Ruiters, Zuyderland Medisch Centrum, Sittard-Geleen; F.J.J. Smeele, Slingeland Ziekenhuis, Doetinchem; Jelle Henk Hofstra, Streekziekenhuis Koningin Beatrix, Winterswijk; Vincent van Driel, HagaZiekenhuis, Sportlaan, Den Haag; Pieter Nierop, Franciscus Gasthuis, Rotterdam; Ward Pieter Josan Jansen, Tergooiziekenhuizen, Hilversum;

Lukas Dekker, Catharina Ziekenhuis Eindhoven, Eindhoven; C.L. Alblas, Franciscus Vlietland, Schiedam; Adrianus Kuijper, Spaarne Gasthuis, Hoofddorp; Ka wai Wu, Van Weel-Bethesda Ziekenhuis, Dirksland; Pieter Hoogslag, Diaconessenhuis Meppel, Meppel; Cornelis De Nooijer, Maxima Medisch Centrum, Veldhoven; Mario Creanza, Beatrix Ziekenhuis, Gorinchem.

Portugal

Filipe Seixo, Centro Hospitalar de Setúbal - Hospital de São Bernardo, Setúbal; Pedro Monteiro, Clínica Cuida Mais – Cuidados de Saúde, Mangualde; Vergílio Schneider, Clínica Vergílio Schneider, Angra do Heroísmo; José António Santos, Centro Hospitalar do Baixo Vouga - Hospital de Aveiro, Aveiro; Carlos Aguiar, Instituto do Coração, Carnaxide; Jorge Martínez, Centro Hospitalar Cova da Beira, Covilhã; Tiago Gregório, Centro Hospitalar de Vila Nova de Gaia/Espinho, Vila Nova de Gaia; Fausto Pinto, Hospital Santa Maria, Lisboa; Eduardo Oliveira, Hospital Lusíadas Lisboa, Lisboa.

Spain

Manuel Almendro Delia, Hospital Universitario Virgen Macarena, Sevilla; Manuel Anguita Sánchez, Clínica Cardiologica Dr. Anguita, Córdoba; Alfredo Palomino García, Hospital Universitario Virgen Del Rocío, Sevilla; Juan José Gómez Doblas, Hospital Universitario Virgen De La Victoria, Málaga; José Javier García Alegría, Hospital Costa Del Sol, Marbella; Gonzalo Baron Esquivias, Hospital Universitario Virgen Del Rocío, Sevilla; Manuel Beltrán Robles, Hospital Virgen Del Camino, Pamplona; Almudena Valle, Hospital Costa Del Sol, Marbella; Jose Maria Fernandez Rodríguez, Hospital Carmen Y Severo Ochoa, Cangas de Narcea; Tomás Ripoll Vera, Hospital Son Llàtzer, Palma de Mallorca; Antonio García Quintana, Hospital Universitario De Gran Canaria Dr. Negrin, Las Palmas de Gran Canaria; Antonio Miguel Barragán Acea, Clinica Vida, Sevilla; Melchor A. Rodríguez

Gaspar, Hospital Universitario De Canarias, San Cristóbal de La Laguna; Manuel Rayo Gutierrez, Clinica Cardiorreal, Ciudad Real; Miguel José Corbi Pascual, Hospital General De Albacete, Albacete; Jose Antonio Nieto Rodríguez, Hospital Virgen De La Luz, Cuenca; Pedro Abizanda Soler, Hospital Perpetuo Socorro, Albacete; Jose Manuel Martín Antorán, Hospital Río Carrión, León; Jose Antonio Lastra Galán, Consulta Privada, Sevilla; Jesús Ignacio Domínguez Calvo, Hospital El Bierzo, Ponferrada; Esther Fernandez Perez, Complejo Asistencial Universitario De León, León; Abel García Del Egido, Complejo Asistencial Universitario De León, León; Carlos Molina Cateriano, Hospital Universitari De La Vall D´Hebron, Barcelona; Pere Domènech Santasusana, Hospital Universitari De Bellvitge, L'Hospitalet de Llobregat; Augusto Ordoñez España, Hospital Del Vendrell, Vendrell; José Mateo Arranz, Hospital De La Santa Creu I Sant Pau, Barcelona; Raúl Lafuente Maqueda, Hospital L´Hospitalet, L'Hospitalet de Llobregat; Román Freixa Pamias, Hospital Moises Broggi, Sant Joan Despi; Alejandro Ponz De Tienda, Hospital Clinico Universitario De Valencia, Valencia; Enrique Santas Olmeda, Hospital Clinico Universitario De Valencia, Valencia; Lorenzo Fácila Rubio, Hospital General Universitario De Valencia, Valencia; Jose Maria Cepeda Rodrigo, Hospital Vega Baja, Alicante; Vicente Bertomeu González, Hospital Universitario San Juan De Alicante, Alicante; Juan Gabriel Martinez Martinez, Hospital General Universitario De Alicante, Alicante; Carlos De Diego Rus, Hospital Universitario De Torrevieja, Torrevieja; Miguel Ahumada Vidal, Hospital General Universitario De Elche, Elche; Josep Navarro Manchón, Hospital General De Castellon, Castellón; María Amparo Albert Contell, Hospital De Sagunto, Sagunto; Francisco Angel González Llopis, Hospital General Universitario Elda, Elda; Inmaculada Castillo Valero, Hospital De Sagunto, Sagunto; Ruth Mª Sánchez Soriano, Hospital Virgen De Los Lirios, Alcoi; Jose Javier Gomez Barrado, Hospital San Pedro De Alcántara, Cáceres; Jose Ramón González Juanatey, Hospital Clinico Universitario De Santiago, Santiago de Compostela;

ACEDO DOMINGUEZ Santiago Castro, Complejo Hospitalrio Universitario A Coruña, A coruña; Maria Ferreiro Argüelles, Complexo Hospitalario De Pontevedra, Pontevedra; Manuel Méndez Bailón, Hospital Clinico San Carlos, Madrid; Mª Isabel Antorrena Miranda, Hospital Universitario La Paz, Madrid; Carlos Álvarez, Hu La Paz, Madrid; Luis Miguel Rincón, Hospital Universitario Ramón Y Cajal, Madrid; Esther Montero Hernández, Hospital Puerta De Hierro, Majadahonda; Joaquín Carneado Ruiz, Hospital Universitario Puerta De Hierro, Majadahonda; Mª Del Mar Contreras Muruaga, Hospital Universitario De La Princesa, Madrid; Felipe Atienza Fernández, Hospital General Universitario Gregorio Marañón, Madrid; Francisco J. Rodríguez Rodrigo, Hospital Universitario Madrid Montepríncipe Centro Integral Enfermedades Cardiovasculares, Boadilla del monte; Natalia Acedo Dominguez, Hospital Universitario De La Princesa, Madrid; Juan L. Rodríguez Calderón, Hospital San Rafael, Madrid; Rafael Salguero Bodes, Hospital Universitario 12 De Octubre, Madrid; Francisco Javier Parra Jiménez, HM Madrid (H. U. HM Madrid, Cardiology, Madrid 3084), Madrid; Julio Ismael Osende Olea, Hospital Universitario HM Sanchinarro, Madrid; Juan Nepomuceno Medina Peralta, Hospital Universitario HM Montepríncipe - Boadilla del Monte, Madrid; Cristina Llanos Guerrero, Hospital Hm Vallés - Alcalá de Henares, Madrid; Eddy Velásquez Arias, Hospital Universitario HM Torrelodones - Torrelodones, Madrid; Jesús Palomo Álvarez, Hospital Universitario HM Puerta Del Sur, Móstoles; Sergio Manzano, Hospital Clínico Universitario Virgen De La Arrixaca, El Palmar, Murcia; Matias Perez Paredes, Hospital General Universitario Morales Meseguer, Murcia; Antonio Javier Trujillo Santos, Hospital Universitario Santa Lucía, Cartagena; Ramón Rubio Patón, Hospital General Universitario Santa Lucia, Cartagena; Nuria Basterra Sola, Complejo Hospitlario De Navarra, Pamplona; José Ignacio García Bolao, Clinica Universitaria De Navarra, Pamplona; Carmen Jimenez, Hospital Universitario De Burgos, Burgos; Emengol Vallese, Hospital Del Mar, Barcelona; Milagros Suito Alcántara, Hospital Universitario De

La Vall D´Hebron, Barcelona; Rebeca Muñumer Blazquez, Hospital El Bierzo, Ponferrada; Victoria Moreno Flores, Hospital De La Vega Baja, Orihuela; Martín Asengo, Hospital Clínico De Valladolid, Valladolid; María Robledo, Hospital Universitario De Álava, Gasteiz; Sebastian Isaza, Hospital Comarcal Medina del Campo, Medina del campo; José González Ruiz, Hospital De Galdakao-Usansolo, Galdakao.

Switzerland

Alexander Breitenstein, Universitaetsspital Zuerich, Zuerich; Thomas Fischer, Kantonsspital Winterthur, Winterthur; Cyril Pellaton, Hôpital neuchâtelois, Neuchatel; Jean-Jacques Goy, Hôpital Fribourgeois - Freiburger Spital, Fribourg; Mehdi Namdar, Geneva University Hospital, Geneva; Stephane Cook, Hôpital Fribourgeois - Freiburger Spital, Fribourg; Pascal Chatelain, Pascal Chatelain Private Practice, Geneva; Christoph Schalcher, Gemeinschaftspraxis Greifensee, Greifensee; Robert Escher, Regionalspital Emmental AG, Burgdorf; Juerg H. Beer, Kantonsspital Baden AG, Baden; Angelo Auricchio, Cardio Centro Ticino, Lugano; Jean-Luc Reny, Hopital Universitaire Geneve, Thônex-Genève; Daniel Nobel, Spital Wil, Wil; Joerg Nossen, Luzerner Kantonsspital, Sursee; Alexander Imhof, Spital Region Oberaargau, Langenthal; Flavio Acquistapace, Studio Cardiologico Acquistapace, Manno; Juan Sztajzel, Clinique de Carouge, Geneva; Giuseppe Cocco, Practice of Dr. med. Giuseppe Cocco, Rheinfelden; Robert Bonvini, Clinique des Grangettes, Chene-Bougeries; Peter Ammann, Kantonsspital St. Gallen, St. Gallen; Flavio Acquistapace, Studio Medico Canepa, Mezzovico; Flavio Acquistapace, Studio Medico Sanacare, Lugano; Jens Hellermann, Facharzt FMH fuer Innere Medizin und Kardiologie, Schiers; Stefan Kradolfer, Facharzt FMH ALLG. Medizin, Basel; Ralf Polikar, Nyon.

United Kingdom

Ahmet Fuat, Carmel Surgery, Darlington; Philip Keeling, Torbay Hospital, Torquay; Ameet Bakhai, Barnet Hospital, Barnet; Terry McCormack, Whitby Group Practice, Whitby; Lawrence Barnes, Rame Medical Limited, Torpoint; Azhar Zafar, Danes Camp Medical Practice, Northampton; Mark Davies, West Cross Medical Centre, Swansea; Michael Butler, Waterloo Medical Centre, Blackpool; Anoop Chauhan, Blackpool Victoria Hospital, Blackpool; Roxy Senior, Northwick Park Hospital, Harrow; Paul Nixon, Swan Lane Medical Centre, Bolton; Matthew Seager, Dr C J C Johns & Partners, Killay; Martin James, Royal Devon & Exeter Hospital, Exeter; Piers Clifford, Buckinghamshire Healthcare NHS Trust, High Wycombe; Andrew Moriarty, Craigavon Area Hospital, Co. Armagh; John Ryan, The Alverton Practice, Penzance; Steven Lindsay, Bradford Royal Infirmary, Bradford; David Neilson, Dumbarton Health Centre, Dumbarton; Savio D'Souza, Darent Valley Hospital, Dartford; Gabrielle Slade, Forest End Surgery, Waterlooville; Scot Garg, Royal Blackburn Hospital, Blackburn; Simon Cartwright, The White Horse Medical Practice, Faringdon; Michael Seddon, Musgrove Park Hospital, Taunton; Honer Kadr, Queen's Hospital, Romford; Roland Veltkamp, IMP COLL HEALTH NHST 1, London; Joanne Porter, Peterborough City Hopsital, Peterborough; Neville Kukreja, Lister Hospital, Stevenage; Patrick Eavis, Heart of Bath Medical Partnership, Bath; Mark Richardson, Lindum Medical Practice, Lincoln; Matthew Fay, Westcliffe Medical Practice, Bradford; Mark Bowman, The Ulster Hospital, Belfast; Nicholas Linker, James Cook University Hospital, Middlesbrough; Azfar Zaman, Freeman Hospital, Newcastle upon Tyne; Sachin Jadhav, Nottingham University Hospitals City Campus, Nottingham; Dhiraj Gupta, Liverpool Heart and Chest Hospital, Liverpool; David MacDougall, Hairmyres Hospital, East Kilbride; Gunaratnam Gunathilagan, Queen Elizabeth the Queen Mother Hospital, Margate; Anna Maria Choy, Ninewells Hospital, Dundee; Mehrdad Malekianpour, University Hospital Wishaw, Wishaw;

Washik Parkar, Gloucester House Medical centre, Urmston; Ravish Katira, St Helens & Knowsley Teaching Hospital NHS Trust, Prescot; Timothy Myhill, Rothwell & Desborough Healthcare Group, Wellingborough; Sarah Maxwell, The Crouch Oak Family Practice, Weybridge; David Jones, Harefield Hospital, Harefield; Conor McCann, Belfast City Hospital, Belfast; Duncan Rogers, Mowbray House Surgery, Northallerton; Craig Barr, Russells Hall Hospital, Dudley; David Hargroves, William Harvey Hospital, Ashford; Fiona Bellas, Lambert Medical Centre, Thirsk; Neeraj Prasad, Wye Valley NHS Trust-County Hospital, Hereford; Jason Glover, Basingstoke and North Hampshire Hospital, Basingstoke; Javed Ahmed, Freeman Hospital, Newcastle upon Tyne; Jeremy Platt, The Binfield Surgery, Binfield; Paula Marrett, The Fowey River Practice, Fowey; Simon Hutchinson, Ballygomartin Group Practice, Belfast; Swaminathan Thiagarajan, Pickering Medical Practice, Pickering; Graeme Little, Sleights and Sandsend Medical Practice, Whitby; Daniel Berkeley, Maryport health services, Maryport; Arnold Berger, Fishermead Medical Centre, Milton Keynes; Damien Cullington, Leeds General Infirmary, Leeds; Manish Saxena, Barts Health, London; Louise Buchanan, North Cumbria University Hospitals NHS Trust, Carlisle; Sam McClure, Sunderland Royal Hospital, Sunderland; Chris Gregory, Royal Albert Edward Infirmary, Wigan; Masood Khan, Watford General Hospital, Watford; Amrit Takhar, Wansford and Kings cliffe practice, Peterborough; Aravindakshan Manoj, Broadgreen Hospital, Liverpool; Charalampos Kartsios, Birmingham Heartlands Hospital, Birmingham; Kneale Metcalf, Norfolk and Norwich Hospital, Norwich; William Francis, Stokesley Health Centre, Stokesley; Philip Campbell, Royal Gwent Hospital, Newport; Anand Dixit, Freeman Hospital, Newcastle upon Tyne.
